# Multiple *Cis-acting* Polypyrimidine Tract Elements Regulate a Cooperative Mechanism for Triticum Mosaic Virus Internal Ribosomal Entry Site Activity

**DOI:** 10.3389/fpls.2022.864832

**Published:** 2022-04-12

**Authors:** Helena Jaramillo-Mesa, Emma Fischer, Aurélie M. Rakotondrafara

**Affiliations:** Department of Plant Pathology, University of Wisconsin-Madison, Madison, WI, United States

**Keywords:** internal ribosome entry site, YX-AUG, polypyrimidine tract, translation initiation, triticum mosaic virus, AUG selection, 40S and 60S ribosomal subunits

## Abstract

Diverse elements within the 5′ untranslated region of an mRNA can influence the translation efficiency at the main AUG codon. We previously identified a core picornaviral like Y_16_X_11_-AUG motif with 16-nt polypyrimidine CU tract separated by an 11-nt spacer sequence from the 13th AUG codon, which is recognized as the preferred initiation site within the *Triticum mosaic virus* (TriMV) internal ribosome entry site (IRES) element. The motif is proposed to function as an internal ribosomal landing site at the designated start codon. Here, we exposed the cooperative role of multiple CU-rich segments flanking the TriMV YX-AUG motif to reach and drive internal initiation of translation at the preferred start site. We propose that these auxiliary domains may enhance the ribosome capacity and their delivery at proximity of the correct initiation site. These polypyrimidine tracts can be modulated with a cryptic AUG in a position-dependent manner to replace the native YX-AUG motif, and thus uncovering a new layer of control of start codon selection. In line with these observations, mass spectrometry analysis of proteins directly interacting with translationally impaired TriMV IRES mutants that bear these motifs indicated an enrichment in 40S and 60S ribosomal related proteins, revealing a new function of polypyrimidine tracts to regulate IRES-driven translation. Accessibility of these RNA regions for *in trans* interaction was validated by SHAPE analysis of the entire TriMV leader sequence and supported by the ability of anti-sense oligonucleotides designed to block the CU tracts accessibility to impair IRES activity. This is the first evidence that defines the core modular domains required for ribosomal recruitment and start codon selection in a complex, multi-AUG viral 5′ UTR for translation in plants.

## Introduction

Translation initiation is a critical process that requires recruitment of the ribosomal complex to the mRNA for the recognition of the initiation site (Hinnebusch and Lorsch, 2012). Eukaryotic translation is mediated by the 5′ m7GpppG cap, which recruits the 43S pre-initiation ribosomal complex composed of the small 40S ribosomal subunit bound to the translation initiation factors eIF1, eIF1A, eIF3, and the eIF2/GTP/Met-tRNA_i_^Met^ ternary complex. The complex initially attaches to the 5′ end of the mRNA and then scans linearly along the 5′ leader region in search for an AUG in a suitable context. Upon the recognition of the correct AUG codon, it forms a 48S initiation complex with established codon–anti-codon base pairing that leads to the release of the inorganic phosphate (Pi), which makes the GTP hydrolysis by eIF2 irreversible, followed by the joining of the large 60S ribosomal subunit to the 48S complex (Hinnebusch and Lorsch, 2012). The resulting 80S complex is then committed to initiate protein synthesis. According to the “first AUG rule,” translation typically initiates at the 5′ proximal AUG triplet (Kozak, 1995).

Many cellular and viral mRNAs bear more than one AUG codon in their 5′ untranslated region (UTR), and specialized strategies for start codon selection are needed in these cases (Firth and Brierley, 2012; Sriram et al., 2018). The best understood of these strategies is leaky scanning, where the first encountered AUG codon (in a “suboptimal” sequence context) remains silent and ribosomes skip to the next downstream AUG (Kozak, 2002; Wang and Rothnagel, 2004). In other cases, ribosomes terminate translation at a small upstream open reading frame (uORF) prior to re-initiating at a downstream “main” AUG site located at a shorter distance (>50 nucleotides in length; Kozak, 2001; Skabkin et al., 2013), or undergo “ribosomal shunting” allowing ribosomal complexes to first translate a short uORF prior to bypassing a large portion of the 5′ UTR through looping to the correct initiation site, often hundreds of bases downstream (Yueh and Schneider, 2000; Sherrill and Lloyd, 2008; Pooggin et al., 2012). Many positive (+)-sense RNA viruses rely on internal ribosomal entry sites (IRES) embedded within their relatively long 5′ UTRs to overcome the requirement for a 5′ cap; a strategy that also allows them to navigate complex 5′ UTRs encoding multiple silent AUG codons (Balvay et al., 2009). During IRES-driven translation, the ribosomal complexes are recruited to an internal RNA structure rather than to the 5′ end and that often positions them at proximity to the preferred AUG codon and thus bypassing a large portion of the 5′ UTR (Balvay et al., 2009). The influence of uAUGs and/or uORFs on the start codon recognition strategy and on the modulation of translation initiation and gene expression has been recognized (Wang and Rothnagel, 2004; Zhang et al., 2020). Even though there is increasing evidence of the high frequency of uAUGs in cellular mRNAs and viral RNA sequences, the mechanistic details of these translation regulation strategies remain largely unknown (Wang and Rothnagel, 2004; Zhang et al., 2020).

In the multi-AUG bearing picornavirus IRESes, the preferred initiation sites seem to be specified through binding of a subset of translation initiation factors and cellular trans-acting factors that promote ribosome complex loading onto a conserved Yn-Xm-AUG motif that often embeds the preferred initiation site, with “Y” being a polypyrimidine-rich tract separated by an Xm spacer with non-specific nucleotide sequence before an AUG codon (Wimmer and Paul, 2010; Lozano and Martinez-Salas, 2015). The two best-characterized YX-AUG motifs are found in type I Poliovirus (PV) motif (Y_9_X_18_-^a^AUG-X_154_-^b^AUG motif) and the type II Encephalomyocarditis virus motif (EMCV; Y_9_X_10_-^a^AUG-X_5_-^b^AUG motif; Hellen et al., 1994; Kaminski et al., 1994). Both YX-AUG types bear a cryptic AUG codon downstream of the polypyrimidine Y tract that is bypassed by the ribosomal complex to initiate translation at a further downstream AUG. These type I and type II motifs differ in the scanning ability of the recruited ribosomal complex to reach that downstream initiation site. In the PV Y_9_X_18_-^a^AUG-X_154_-^b^AUG motif, the authentic polyprotein ^b^AUG codon is located 154 bases further downstream of the cryptic ^a^AUG triplet and likely reached by ribosomal scanning mechanism (Hellen et al., 1994; Pestova et al., 1994). For the EMCV Y_9_X_10_-^a^AUG-X_5_-^b^AUG motif, the ribosomal complex initiates at the downstream ^b^AUG positioned at a much shorter distance from the CU-rich tract (18 nts) and from the cryptic ^a^AUG (5 nts; Ohlmann and Jackson, 1999). In this context, a long spacer sequence resulted in poor translation of the downstream AUG, revealing the relevance of a “good” spacer length for the recognition of the proper initiation site (Hellen et al., 1994; Pestova et al., 1994). Moreover, while it is not utilized as a start codon, the cryptic uAUG is an essential component of the YX-AUG motif. Mutation or deletion of the codon was reported to shift the recognized start site (Ohlmann and Jackson, 1999).

In addition to the YX-AUG motif that helps to designate the ribosomal entry site, another key feature of picornavirus IRESes is the presence of additional polypyrimidine-rich tracts within their sequences that act as cis-acting regulatory elements. In fact, these CU-rich regions are reported to provide binding sites for the polypyrimidine tract binding protein (PTB), which stimulates IRES activity by stabilizing the IRES structure (Kafasla et al., 2009; Martinez-Salas et al., 2017). These CU-rich regions have been suggested as potential different roles in some plant RNA viruses. The CU-rich motifs have been shown to be essential for cap-independent translation by exhibiting direct complementarity with a conserved region of the 18S rRNA, as reported for the *Tobacco etch virus* (TEV) CIRE (Cap-independent regulatory element; Zeenko and Gallie, 2005), the *Blackcurrant reversion virus* (BRV) IRES elements (Karetnikov and Lehto, 2007), and the 19 nt CU-rich region of *Turnip crinkle virus* 5′ UTR (Stupina et al., 2011). Although PTB homologs have been found in plants, their role in cap-independent translation has not been explored yet (Ruhl et al., 2012).

*Triticum mosaic virus* (TriMV), a member of the largest plant virus family *Potyviridae*, is remarkable in that it bears an exceptionally long (739 nucleotides) 5′ UTR that contains 13 AUG triplets, with the 13th AUG serving as the designated start codon (Seifers et al., 2008; Tatineni et al., 2009; Zhang et al., 2015). The genome of TriMV is a polyadenylated positive-sense RNA of about 10,200 nucleotides, has a 48.6 kDa viral protein linked to the genome (VPg) at its 5′ end that replaces the 5' cap, and encodes a single polyprotein (Seifers et al., 2008; Tatineni et al., 2009). Our group demonstrated that the TriMV 5′ UTR bears a complex IRES (Roberts et al., 2015, 2017a) with a core Y_16_X_11_-AUG_740_ motif embedding the preferred AUG codon at position 740 and an 11-nt X spacer length (Jaramillo-Mesa et al., 2019). This TriMV YX-AUG motif can be functionally replaced with variants of either type I PV or type II EMCV motifs, revealing strong conservation of function for both plant and mammalian viruses for ribosomal entry site (Jaramillo-Mesa et al., 2019). We have shown that, similar to the EMCV YX-motif (Hellen et al., 1994; Kaminski et al., 1994), a short X spacer ( ≤ 11 nts) is vital to the integrity of the TriMV YX-AUG function, in line with a model where the TriMV 5′ UTR-mediated translation initiates at the YX-AUG embedded start codon reached without scanning. While we have yet to show a direct physical interaction, the TriMV Y_16_X_11_-AUG_740_ motif contains two 10-nt and 7-nt long binding sites exhibiting complementarity to a G-rich stretch within a highly conserved region of the 18S rRNA. Mutations that disrupted overall complementarity to the 18S rRNA and delivery of anti-sense oligonucleotides designed to block the YX-AUG accessibility impaired IRES activity (Jaramillo-Mesa et al., 2019).

Sequence analysis revealed that the YX-AUG-like motifs might be a conserved feature of the 5′ UTRs of virus members of the *Potyviridae* family with multiple AUGs (Jaramillo-Mesa et al., 2019). However, the contribution of the YX-AUG motif to IRES-driven translation may likely be dependent on additional cis-acting regulatory elements. In fact, the presence of an YX-AUG on these other potyviral 5′ UTRs is insufficient to support IRES activity (Jaramillo-Mesa et al., 2019). Herein we describe a new role for polypyrimidine-rich tracts as cis-acting regulatory element of IRES activity in plant viruses. We revealed that the TriMV Y_16_X_11_-AUG_740_ motif requires two additional flanking CU-rich tracts located on a large stem structure at upstream positions to cooperatively drive translation at the correct AUG. Mass spectrometry analysis of proteins interacting with these motifs indicated an enrichment in 40S and 60S ribosomal related proteins, which suggests a role in locally clustering ribosomal complexes at the 3′ end of the IRES. Embedding an AUG codon within these auxiliary CU-rich segments to create chimeric YX-AUG motif was sufficient to reprogram translation initiation of translationally impaired mRNAs. Remarkably, we unraveled that the distance separating the CU-rich tract from the first encountered AUG codon specifies ribosome processivity and selection of the favorable initiation site. This is the first evidence that translation can be reprogrammed using chimeric YX-AUG-like motifs and helps define the core requirements for ribosomal recruitment and start codon selection in a complex, multi-AUG viral 5′ UTR.

## Materials and Methods

### Luciferase Reporter Constructs

All of the TriMV cDNA clones in the monocistronic constructs with the stable hairpin at the 5′ end of the untranslated region were generated as described previously (Roberts et al., 2015). The monocistronic TriMV firefly luciferase constructs were assembled in the T3 polymerase-driven plasmid, c-myc-T3LUC(pA; Thoma et al., 2004). The TriMV mutated sequences were synthesized as gBlocks^®^ fragment from IDT-DNA or as GenParts from GenScript and next cloned using the recombination-based cloning kit from GenBuilder from GenScript into the TriMV plasmid cut at HpaI-NcoI sites. The sequence inserted at the very 5′ end of the untranslated region in each clone, to form a stable stem loop with a ΔG > −34 kcal (Kozak, 1989), was CGC GCG CAC GGC CCA AGC TGG GCC GTG CGC GCC.

### Transcription

All RNAs were transcribed *in vitro* from linearized plasmids or PCR-amplified products using either the T7 MegaScript kit from Ambion or the T3 RNA polymerase from Thermo Fisher. Monocistronic TriMV luciferase constructs and the control firefly luciferase construct with vector sequences were linearized either with SfcI/BfmI to include the poly(A) tail. The Renilla luciferase construct (Promega) using for the *in vivo* assays was linearized with BamHI to include the poly(A) tail. Reactions were assembled according to the appropriate transcription kit protocol and as previously described (Roberts et al., 2015).

### Translation Assays and Data Collection

The *in vitro* translation reactions were performed using the wheat germ extract system kit (Promega, Madison, WI) and as previously described (Roberts et al., 2015). All experiments were performed in triplicate and repeated in at least three independent experiments. For the translation assays in the presence of the anti-sense DNA oligos, luciferase activity was measured for the full-length TriMV 5′ UTR mRNA construct with the strong stem loop at the immediate 5′ end. The translation reaction was set up as previously described (Zeenko and Gallie, 2005) except the addition of magnesium acetate at a final concentration of 0.625 mM to rule out any loss of translation due to magnesium chelation by the DNA oligos. The unmodified DNA oligonucleotides were ordered at IDT-DNA. The sequences were:

Anti YX-AUG: 5′AGGAGAAAAAGAGAAGTA 3′.

Anti-Beta-globin: 5′ CAAAAGCTTGCAGGAAGAGATCCATCTAC 3′.

Anti Y-1: 5′ GAGAATAGTGGAAAAACAGGGA 3′.

Anti Y-2: 5′ AAAGTAAGAAAGTGCCAAGAGAGCAGAAAG 3’.

### *In vivo* Translation in Oat Protoplasts

Oat protoplasts were prepared from an oat cell suspension culture as described (Roberts et al., 2015, 2017b). 1 pmol of each RNA reporter construct was electroporated into approximately 10^6^ cells. To normalize RNA incorporation into cells, 0.1 pmol of capped polyadenylated Renilla luciferase RNA was included in each electroporation. Five hours post-electroporation, the cells were harvested, lysed in 500 μl passive lysis buffer (Dual Luciferase kit, Promega), and centrifuged for 10 min at 15,000 × *g*. Luciferase activity was measured using 100 μl of the supernatant. 50 μl of the luciferase assay reagent was injected into each sample and read for 10 s. 50 μl of Stop & Glo reagent was then injected and Renilla luciferase activity was measured for six seconds. All experiments were performed in triplicate and repeated in at least three independent experiments.

### Selective 2′ Hydroxyl Acylation Analyzed by Primer Extension

SHAPE analysis was conducted as described by Liu et al. (2021). In summary, 8 pmol of *in vitro* RNA previously transcribed as explained above, were diluted to 30 ul of water and denatured at 95°C for 2 min, following a 2 min ice incubation. 7.5 ul of 5X RNA folding buffer (1X folding buffer = 100 mM HEPES pH 8.0, 50 mM MgCl_2,_ 100 mM NaCl) was added to each sample, and samples were incubated at 30°C for 20 min. Each sample was divided in two tubes. To one of the divided reactions, 3.3 μl of 100 mM NMIA was added, and to the other reaction 3.3 μl of DMSO was added. Samples were incubated at 37°C for 45 min. After modification, the RNA was precipitated with 0.1 volumes of 3 M sodium acetate, 2.5 volumes of cold ethanol, and 0.5 μl of 20 mg/ml glycogen. RNA was refrigerated at −80° C for 30 min and centrifuged for 30 min at 4°C. The pellet was washed twice with cold 70% ethanol and air-dried at room temperature for 5 min, the pellet was reconstituted in 10 μl of 0.5X TE buffer. Integrity of RNA was verified in a 1% TBE agarose gel. For cDNA synthesis, 2 pmol of the control (DMSO), modified (NMIA) RNA, or unmodified RNA (for sequencing ladder) were diluted to 8 μl of 0.5X TE buffer. 1 μl of 10 μM of fluorescently labeled primer (Thermo Fisher, 6FAM was used for DMSO and NMIA samples, and PET was used for sequencing ladders) was added to the DMSO and NMIA reactions. Samples were incubated at 65°C for 5 min and placed in ice for 5 min. After incubation, 8.5 μl of enzyme mix (4 μl 5X SuperScriptIII FS buffer, 1 μl 100 mM DTT, 1 μl 10 mM dNTPs, 2ul H_2_O, and 0.5 μl SSIII enzyme) were added (for the sequencing reaction, 2 μl of 1 mM ddATP were also added to the mix). Samples were incubated at 52°C for 1 h and 1 μl of 4 M NaOH was added to the samples to remove the RNA after a 5 min incubation at 95°C. The reaction was neutralized with 2 μl of 2 M HCl and incubated at 95°C for 5 min. Each NMIA and DMSO reactions were combined individually with the sequencing reaction. cDNA was precipitated as described above and resuspended in 20 μl of H_2_O. Samples were sent to Genewiz for fragment analysis. Data were analyzed using QuSHAPE software (Karabiber et al., 2013) were peak position were manually adjusted. Three replicates were submitted, and the procedure was repeated three times. After obtaining the reactivity data, secondary structure analysis prediction was performed using the RNAstructure software from the Mathews Lab (Reuter and Mathews, 2010). Primer sequences used: TriMV (798–818) = 5′ TGCTCTCCAGCGGTTCCATCC 3′ and TriMV (834–854) = 5′ GGAACCAGGGCGTATCTCTTC 3′.

### Pull-Down Assay and Mass Spectrometry Analysis

5′ UTR RNA fragments were synthesized *in vitro* by first linearizing the firefly luciferase construct above mentioned with BbsI to remove the luciferase coding region and produce only 5′ UTR segments. The digestion fragment was then *in vitro* transcribed using the transcription protocol described above. The RNA fragments bear a stable stem loop structure at their 5′ end and were biotinylated at the 3′ end using the Pierce RNA 3′ End Desthiobiotinylation kit from Thermo Fisher (Product No. 20163) and following the manufacturer’s instructions. After biotinylation, RNA was cleaned using the NucleoSpin RNA Clean-up kit from Macherey-Nagel (Product No. 740948.50) and RNA integrity was verified in a 1% agarose gel. 100 μl translation reactions were set up using wheat germ extract system kit (Promega, Madison, WI) with 50 μl of wheat germ extract, 8 μl of amino acid mix, 8 μl of 1 M potassium acetate, 4 μl of 50 mM of GMP-PNP, and 25 pmol of labeled RNA. Reactions were incubated for 10 min at 25°C and UV crosslinked using 254 nm bulbs, for 45–60 s using the auto-crosslink function. After crosslinking, a pull-down of bound proteins was conducted using the Pierce Magnetic RNA-Protein Pull-Down Kit (Product No. 20164) using the manufacturer’s instructions. After incubation with streptavidin beads, 5 washes were preformed prior to the final elution, to remove all unbound proteins and reduce the occurrence of unspecific bounded proteins. An aliquot of the final elution samples was loaded in an SDS-PAGE and revealed with silver staining to verify protein presence (Supplementary Figure 3A). Remaining final elution was sent to the Mass Spectrometry Core Facility at the University of Wisconsin-Madison for Nano Liquid chromatography with tandem mass spectrometry (LC–MS–MS) analysis. Resulting data were used to search against the *Triticum aestivum* (130,673 entries) UniProt reference proteome (Proteome ID UP000019116) and results were analyzed using the Scaffold software (Proteome Software, Inc). The mass spectrometry analysis was performed on two independent experiments. The mass spectrometry proteomics data have been deposited to the ProteomeXchange Consortium *via* the PRIDE (Perez-Riverol et al., 2022) partner repository and available with the dataset identifier PXD031443.

## Results

### Identification of Upstream, Auxiliary Y-Tract Motifs Required for TriMV IRES Activity

**Figure 1:**
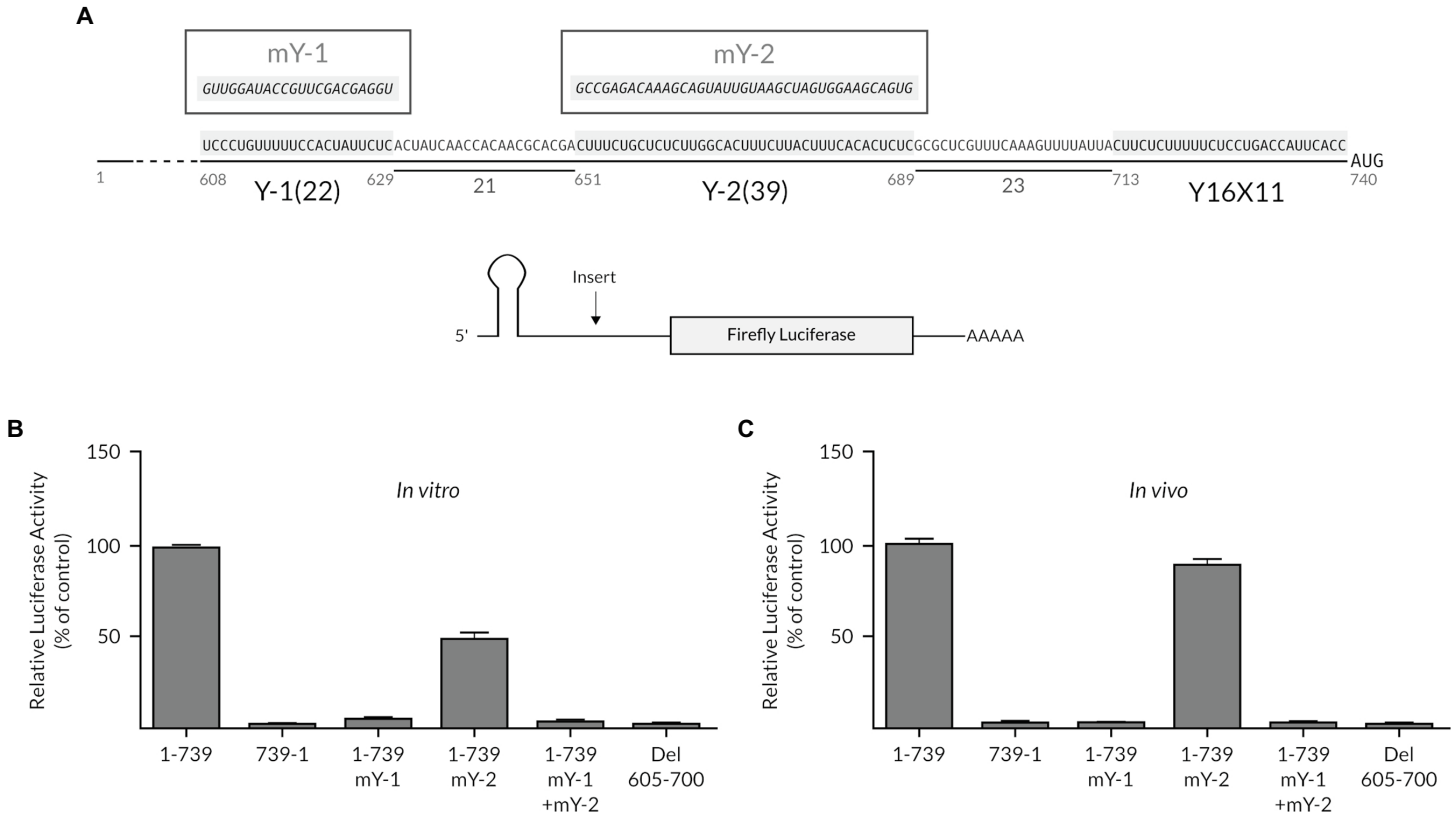
The extended polypyrimidine tracts are key for TriMV IRES activity. **(A)** Sequence of the TriMV 5′ leader (GenBank accession number FJ669487) spanning region nts 608–740, showing the previously described Y_16_X_11_-AUG_740_ motif (Jaramillo-Mesa et al., 2019), and the two flanking (Y-1 and Y-2) polypyrimidine-rich segments at positions nts 608–629 and nts 651–689, with 22-nt and 39-nt segments, respectively. Shown in boxes are the sequences of the mutated Y tracts (mY-1 and mY-2). **(B)** The relative luciferase activities in wheat germ extract of the TriMV 5′ UTR mutants relativized to that of the TriMV wild-type sequence (1-739 construct). **(C)** The relative luciferase activity in oat protoplasts of the reporter mRNAs containing the full-length TriMV leader (1-739) or the various 5’ UTR mutants, normalized to values for a m7GpppG capped polyadenylated Renilla mRNA used as an internal control. In all assays, as a control, we included the full-length TriMV leader sequence (1-739) and the non-functional TriMV reverse complementary sequence (739-1). All TriMV leader sequences were tested in the monocistronic firefly luciferase reporter RNA containing a stable hairpin insertion (delta G = −34 kcal) immediately at the 5′ end of the mRNA (Roberts et al., 2015).

Based on the model that an AUG codon, which is preceded by a polypyrimidine CU-tract, can serve as the ribosomal entry site (Jackson and Kaminski, 1995), we examined the TriMV 5′ untranslated region (UTR) primary sequence surrounding the 12 upstream AUG triplets for additional potential ribosomal landing pads. No CU-rich pattern was observed embedded within or in close proximity to any of these triplets. However, we identified two fairly long CU-rich tracts (22 and 39 bases, respectively) at position nts 608–629 (UCCUgUUUUUCCaCUaUUUCUC) and at nts 651–689 (CUUUCUgCUCUCUUggCaCUUUCUUaCUUUCaCaCUCUC), just upstream of the core TriMV Y_16_X_11_-AUG_740_ region, which embeds the preferred AUG initiation site at position nts 740–742 (Figure 1A). The two polypyrimidine tracts were spaced from one another and from the core Y_16_X_11_-AUG_740_ motif by 21 nucleotides and 23 nucleotides, respectively. Sequence alignments of 12 newly identified TriMV isolates (Redila et al., 2021) revealed the strong sequence conservations of those motifs (Supplementary Figure 1).

We first tested the relevance of these polypyrimidine tracts, which we referred to as Y-1 and Y-2 motifs, respectively, on the IRES-driven translation at the preferred 13th AUG codon. For this purpose, we altered each of the polypyrimidine segments with random sequences in the context of the full-length 739-nts long TriMV 5′ UTR (mY-1*: GUUGGAUACCGUUCGACGAGGU*; mY-2: *GCCGAGACAAAGCAGUAUUGUAAGCUAGUGGAAGCAGUG*, *respectively*; Figure 1A). The TriMV 5′ UTR mutants were tested in the context of a monocistronic *luciferase* reporter mRNA with a stable hairpin structure at the 5′ end to block 5′ entry of the ribosomes and thus ensure measurements of internal (cap-independent) initiation activity (Roberts et al., 2015; Jaramillo-Mesa et al., 2019). In all of these constructs, the core Y_16_X_11_-AUG_740_ motif remained intact with the 13^th^ AUG triplet corresponding to that of the luciferase reporter gene. We compared the translation efficiency of the mutants to that of the wild-type full-length TriMV 5′ UTR (construct 1-739) and the non-functional TriMV reverse complementary sequence (construct 739-1) in two well-validated and complementary plant translation read-outs: wheat germ extract (*in vitro*; Figure 1B) and oat protoplasts (*in vivo*; Figure 1C). Our assay revealed that mutation of the 22-nt long Y-1 sequence (mY-1) abolished translation of the luciferase construct *in vitro* and *in vivo*, while the full sequence disruption of the 39-nt long Y-2 stretch (mY-2) dropped translation down to 50% of the wild-type activity *in vitro* but retained up to 85% of the wild-type level *in vivo* (Figures 1B,C). Mutants with both auxiliary CU-rich motifs swapped into random sequences (1-739: mY-1 + mY-2 mutant) or with their internal deletion (construct del 605–700) were translationally inactive *in vitro* and *in vivo* (Figures 1B,C). These initial observations suggest that these motifs, especially the Y-1 segment, might be necessary for the TriMV IRES-driven translation.

### The Auxiliary Y-Tract Motif Can Be Reprogrammed to Functionally Act as a YX-AUG-Like Motif

**Figure 2:**
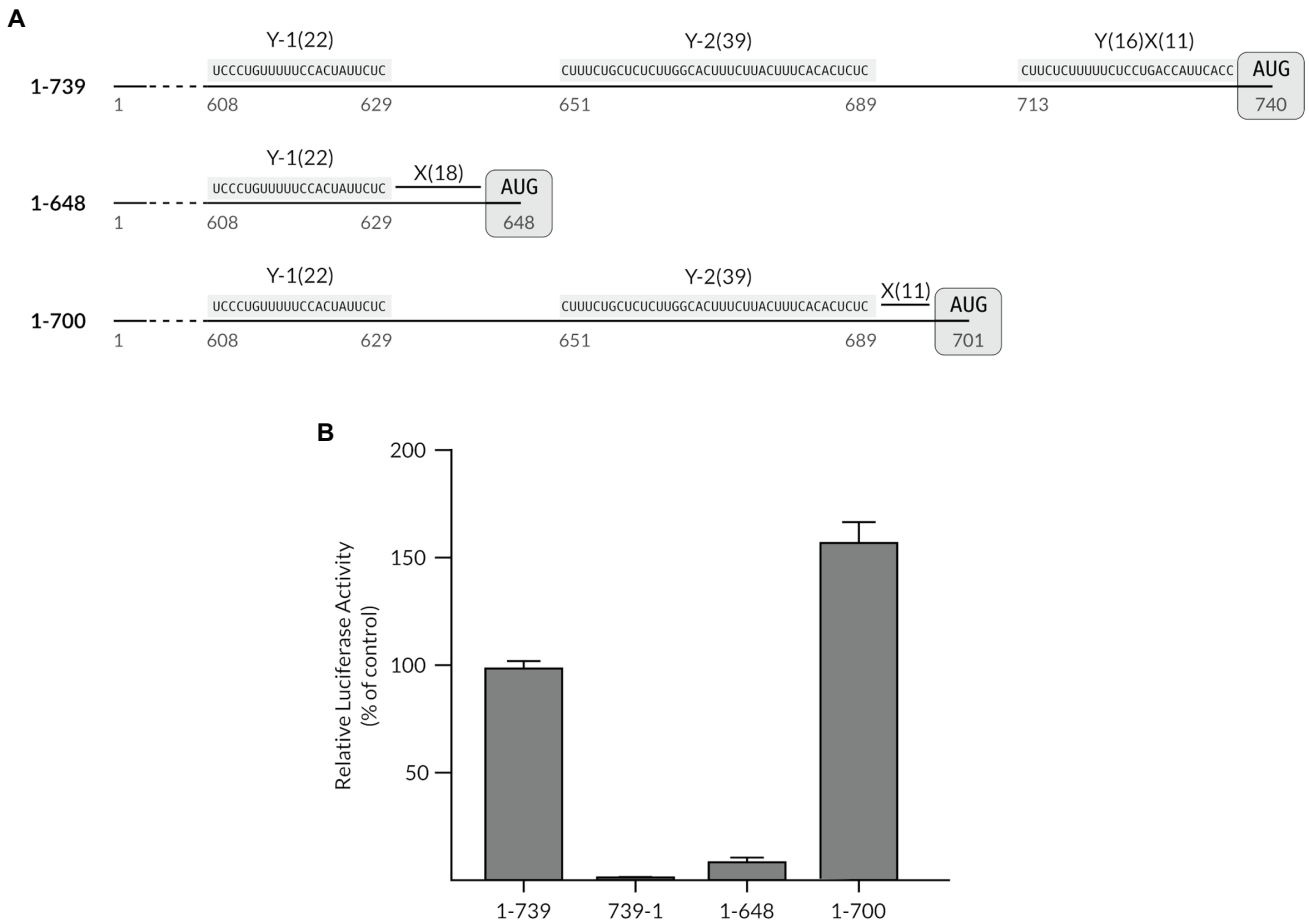
The auxiliary Y-tract can be reprogrammed to act as a YX-AUG-like motif. **(A)** Schematic diagram of the TriMV 5′ UTR deletion mutants compared to the wild-type sequence which bears the Y-1, the Y-2 and the YX-AUG motifs (construct 1-739). Mutant 1-648 was created by deleting region nts 647–740 and positioning the luciferase start codon 18 nt downstream of Y-1 motif at position 648. Mutant 1-700 was created by deleting region nts 701–740 and positioning the luciferase start codon 11 nt downstream of the Y-2 motif at position 701. All deletion mutants are missing the native YX-AUG motif. **(B)** The relative luciferase activities in wheat germ extract of the TriMV 5′ UTR deletion mutants relativized to that of the TriMV wild-type sequence (1-739 construct). The non-functional TriMV reverse complementary sequence (739-1) is added as a negative control.

The sequence patterns of the Y-1 and Y-2 polypyrimidine stretches shared a striking resemblance to a Y_n_X_m_-AUG like motif that is used as a ribosome landing site, (Y_n_ being a stretch of n C and U bases followed by a spacer X of m random bases), except that they did not embed any AUG triplets in their direct close proximity. We have previously shown that deletion of the region spanning the TriMV Y_16_X_11_-AUG_740_ at position nts 709–739 (mutant 1-709) was sufficient to impair IRES-driven translation, revealing the TriMV translation dependency to an YX-AUG motif (Jaramillo-Mesa et al., 2019). The core TriMV Y_16_X_11_-AUG_740_ motif consists of a 11-nt X spacer between the CU tract and the preferred AUG codon at position 740. This preferred AUG codon is located 111 bases and 51 bases downstream of each of the identified Y-1 and Y-2 CU-rich tracts, respectively. Based on the model that an AUG codon embedded within a YX-AUG motif can serve as the ribosomal entry site (Jackson and Kaminski, 1995), we set to test whether these auxiliary polypyrimidine segments and their partial resemblance to a conventional Y_n_X_m_-like motif could functionally reprogram translation. For this purpose, we first truncated the 739-nts long TriMV 5′ UTR to remove the core Y_16_X_11_-AUG_740_ motif, positioning the initiation site of the *luciferase* reporter gene after the Y-1 segment at position nts 648 (mutant 1-648) or after the Y-2 tract at position nts 701 (mutant 1-700; Figure 2A). In these contexts, the AUG triplet was separated by 18 and 11 nucleotides from the closest CU-rich tract, respectively (Figure 2A), resulting in a “Y_22_X_18_-AUG_648_” like motif in the mutant 1-648 construct, and a “Y_39_X_11_-AUG_701_” like motif in the mutant 1-700 RNA. We compared the translation efficiency of these truncated mutants *in vitro* to that of the wild-type full-length TriMV 5′ UTR (construct 1-739) and the non-functional reverse complementary sequence (construct 739-1). While the 1-648 deletion mutant was barely functional, with less than 10% translation activity remaining, the 1-700 deletion mutant strongly supported translation to level well above of the wild-type sequence (Figure 2B).

### The YX-AUG-Driven Translation Is Positionally Dependent and Can Be Modulated by a Cryptic AUG

The above observations led us to examine the requirement by which an YX-AUG motif specifies the translational start site. Of particular focus was the TriMV deletion mutant 1-709 in which the 5′ UTR sequence lacks the native Y_16_X_11_-AUG_740_ but bears intact Y-1 and Y-2 CU-rich segments with the Y-2 polypyrimidine stretch located 20 bases upstream of the AUG, creating a potential Y_39_X_20_-AUG like motif. However, its translation is severely impaired (Jaramillo-Mesa et al., 2019).

**Figure 3:**
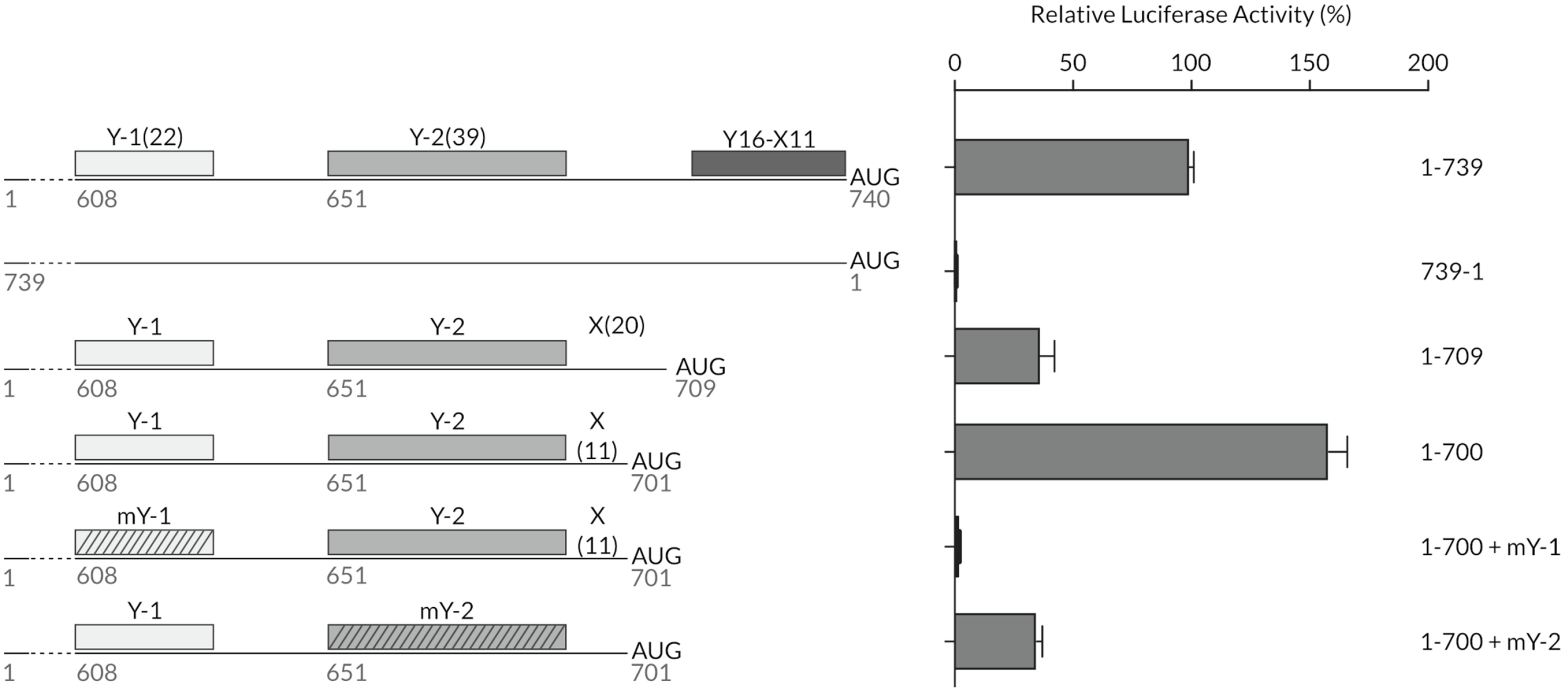
The designation of the correct initiation site is determined by the presence of flanking polypyrimidine tracts and the spacer length (X) of the YX-AUG motif. Schematic diagram of different mutants with their relative luciferase activities in wheat germ extract relativized to that of the wild-type sequence (construct 1-739). The constructs tested consisted of the truncated 1-700 mutant, where the start AUG was placed 11 nt after the Y-2 region creating a Y_39_X_11_-AUG_701_ motif, and where the Y-1 or Y-2 were mutated to random sequences. Constructs were compared to the non-functional TriMV reverse complementary sequence (739-1) and the previously described non-functional mutant 1-709 which lacks the native Y_16_X_11_-AUG_740_ but bears intact Y-1 and Y-2 CU-rich segments with the Y-2 polypyrimidine stretch located 20 bases upstream of the AUG, creating a potential Y_39_X_20_-AUG like motif (Jaramillo-Mesa et al., 2019).

We first revisited the importance of the length of the spacer sequence between the CU-tract and the AUG. We compared the translation efficiency of the 1-700 truncated mutant *in vitro* to that of the wild-type full-length TriMV 5′ UTR (construct 1-739), the non-functional reverse complementary sequence (construct 739-1), and the IRES-impaired deletion mutant (construct 1-709; Jaramillo-Mesa et al., 2019; Figure 3). The 1-700 and 1-709 deletion mutants both lack the native YX-AUG motif and differ in that the AUG codons were positioned 11 and 20 bases downstream of the Y-2 segment, respectively. While 1-709 deletion mutant had 30% of translation activity remaining, the 1-700 deletion mutant strongly supported translation (Figure 3). This observed translation was dependent upon the integrity of both auxiliary CU-rich segments within that region. Altering the 1-700 mutant’s Y-2 tract sequence to random sequences (1-700: mY-2) dropped translation to 30% of wild type, similar to the 1-709 mutant, in line with the requirement of a functional YX-AUG motif for translation; mutating the Y-1 segment (1-700: mY-1) abolished translation. Taken together, the YX-AUG-driven translation is i) dependent upon a “good context spacer length,” likely due to limited scanning ability of the recruited ribosomes as we previously reported for the native TriMV YX-AUG motif (Jaramillo-Mesa et al., 2019) and as reported for the type II EMCV motif (Ohlmann and Jackson, 1999); and ii) dependent on the presence of an additional flanking polypyrimidine track. This is in line with the observed inability of the 1-648 deletion mutant, in which an additional CU-rich segment was missing, to drive translation (Figure 2B).

**Figure 4:**
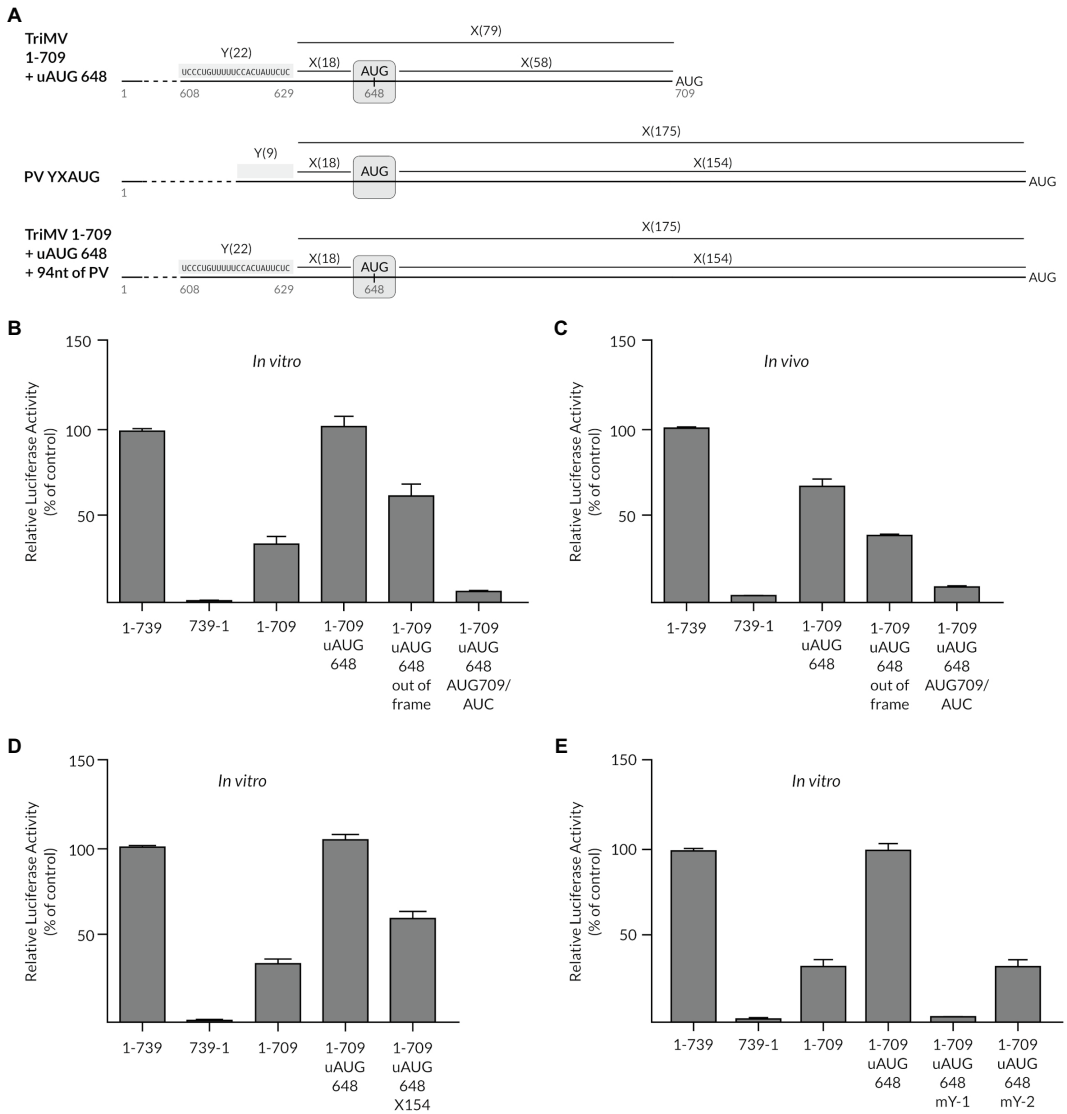
The presence of a cryptic AUG can modulate the YX-AUG-driven translation by likely influencing the scanning ability of the ribosome. **(A)** Schematic diagram of the chimeric YX-AUG constructs created by inserting an upstream AUG at position 648 (uAUG_648_)_,_ 18 nucleotides downstream of the TriMV Y-1 region, in the context of the 1-709 poorly functional mutant described before (Jaramillo-Mesa et al., 2019). The uAUG_648_ is placed in a similar coding frame and Kozak sequence context of the downstream AUG_709_ codon of the luciferase gene. The wild-type Poliovirus Y_9_X_18_-^a^AUG-X_154_-^b^AUG motif is shown for comparison where the ^a^AUG is cryptic and the ^b^AUG is the initiation site. **(B)** Relative luciferase activity of the 1-709 + uAUG_648_ mutants in wheat germ extract relativized to that of the wild-type sequence (construct 1-739). The mutants include the uAUG648 being placed out of frame of the downstream luciferase start codon (construct 1-709 + uAUG_648_), and the luciferase start codon (AUG_709_) being replaced with a non-start AUC codon (construct 1-709 + uAUG_648_ AUG_709_/AUC), to demonstrate the cryptic nature of the inserted uAUG_648_. **(C)** The relative luciferase activity in oat protoplasts of the mutants described in **(B)**, normalized to values for a m7GpppG capped polyadenylated Renilla mRNA used as an internal control. **(D)** The relative luciferase activity in wheat germ extract of the TriMV 1-709 derived mutants with an extended spacer sequence between the upstream uAUG and that of the luciferase gene from X-58 into X-154. The inserted 93 nucleotides corresponded to sequences within the X-154 spacer of the Poliovirus IRES at nts 652–741 (NC_002058.3). **(E)** The relative luciferase activity in wheat germ extract of the TriMV 1-709 + uAUG_648_ derived mutants, with mutants with Y-1 or Y-2 regions mutated to random sequences as described in Figure 1. In all assays, as a control, we included the full-length TriMV leader sequence (1-739) and the non-functional TriMV reverse complementary sequence (739-1).

Secondly, we examined whether the presence of a cryptic AUG could modulate the YX-AUG-driven translation in plants in a similar fashion as reported in both Type I and Type II animal picornavirus IRESes (Hellen et al., 1994; Kaminski et al., 1994). We thus inserted an upstream AUG triplet at position nt 648 (uAUG_648_), 18 nts downstream from the Y-1 motif in the context of the translationally impaired 1-709 deletion mutant (construct 1-709+ uAUG_648_, Figure 4A). This created a chimeric “Y_22_X_18_-^a^AUG-X_58_^b^AUG” motif that imitated the Type I PV Y_9_X_18_-^a^AUG-X_154_-^b^AUG motif, in which the first ^a^AUG triplet is located 18 nts from the Y motif and separated from a downstream ^b^AUG codon by a long spacer sequence, which included the Y2 CU-rich tract (Hellen et al., 1994; Pestova et al., 1994). In the context of the Type I PV YX-AUG motif, the downstream AUG is designated as the initiation site and likely reached by ribosomal scanning (Hellen et al., 1994; Pestova et al., 1994). The inserted uAUG_648_ codon in the TriMV IRES was placed in a similar coding frame and Kozak sequence context of the downstream AUG_709_ codon of the luciferase gene. Remarkably, embedding that AUG right after the Y-1 tract fully restored translation of the deletion mutant 1-709 up to the wild-type level *in vitro* (constructs 1-709+ uAUG 648, Figure 4B). When we placed the uAUG_648_ codon out of frame of the luciferase initiation site (1-709+ uAUG648 out of frame), translation of the luciferase was maintained up to 70% of the full-length wild-type level *in vitro* (Figure 4B). This revealed that the inserted uAUG remained cryptic, as intended, and the downstream AUG_709_ remained the targeted start codon. In line with such a model, when we mutated the AUG_709_ codon of the luciferase into a non-initiation AUC site, we abolished translation in the context of the in-frame chimeric YX1-AUG_648_ construct (construct 1-709 + uAUG_648_ + AUG_709_/AUC; Figure 4B) further supporting the cryptic, but essential nature of the inserted AUG in restoring translation of 1-709 deletion mutant. Similar trend in translation was replicated *in cells* (Figure 4C).

To support the scanning ability of the ribosomal complex that landed on this chimeric YX-AUG_648_ motif to reach the downstream AUG_709_ codon, we extended the spacer sequence separating the two AUG codons from X_61_ to X_154_ to similar length of that in the PV motif “Y_22_X_18_-^a^AUG-X_154_^b^AUG” (Figures 4A,D). For this purpose, we inserted 93 bases from the native PV spacer sequence. The relative frequency of initiation at this new position of the start codon was 70% of the wild-type level *in vitro* (Figure 4D), in line with a scanning-mediated ribosomal mechanism.

This observed translation reprogramming was dependent upon the Y-1 and Y-2 segments (Figure 4E). Swapping the Y-1 CU-rich tract with random sequences, and thus eliminating the chimeric YX-AUG motif, abolished the recovered translation (1-709: uAUG 648 + mY-1 construct, Figure 4E). Altering the Y-2 tract sequence to random sequences (1-709 uAUG 648 mY-2) dropped translation to 30% of wild type, similar to the 1-709 mutant, in line with the requirement of an intact YX-AUG motif and an auxiliary CU-rich tract for activity (Figure 4E).

All together our data supported that the assembly of the ribosomal complex and translation initiation can be reprogrammed at each of these upstream CU-rich tracts under specific conditions: the YX-AUG-driven translation is positionally dependent with the critical role of the spacer length separating the preferred AUG and the CU-rich tract, can be modulated by a cryptic AUG codon, and requires a flanking polypyrimidine track for its function.

### The Flanking Polypyrimidine Segments Are Positioned in Unconstrained Structures in Line With Their Accessibility for Potential Interactions

**Figure 5:**
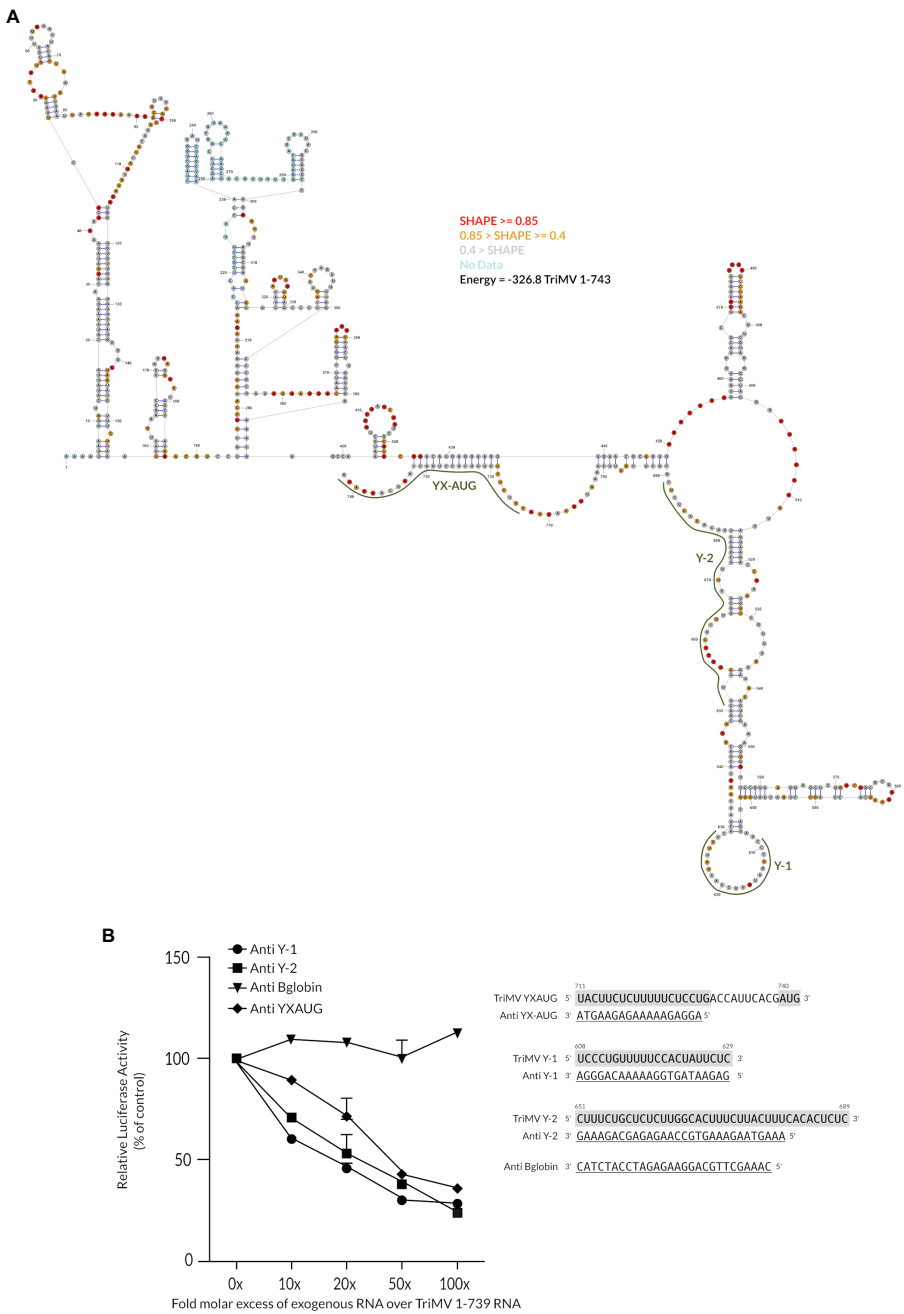
The flanking polypyrimidine segments are positioned in unconstrained structures and their accessibility is essential for translation initiation. **(A)** SHAPE-guided lowest free energy secondary structure of the TriMV 5′ leader (GenBank accession number FJ669487). Y-1, Y-2 and YX-AUG motifs are highlighted in green. Structure was computed using the RNAstructure from the Mathews Lab. Red bases represent SHAPE reactivity data ≥0.85, orange bases 0.85 > SHAPE ≥0.4, gray bases SHAPE reactivity data <0.4, and blue bases represent positions where no SHAPE data was obtained. **(B)** Trans-inhibition assay of the wild-type TriMV 5′UTR RNA with increasing molecular excess of anti-sense single stranded DNA oligonucleotides targeting the Y1-segment (anti Y-1) and the Y-2 segment (anti Y-2) in wheat germ extract. As a control, DNA oligonucleotides targeting the Y_16_X_11_-AUG_740_ motif and unrelated human beta-globin sequence were added. A 0- to 100-fold molar excess of the anti-sense oligonucleotides were added to the reaction.

The above observations led us to hypothesize that the Y-tract regions at upstream positions of the native YX-AUG motif may be the initial recruitment site of the ribosomal complexes onto the TriMV IRES element. To characterize the accessibility of the Y tract regions for such potential interaction, we mapped the overall secondary structure of the 739-nt TriMV 5′ UTR by SHAPE analysis (Figure 5A). The SHAPE-guided structure of the TriMV leader sequence revealed a compact structure with the configuration of the 3′ end of the UTR comprising two defined stem structures, which meet at a three-way junction with the YX-AUG motif located on a downstream unstructured segment. These stems correspond to the hairpin at position nts 469–488 that was previously identified to be crucial for eIF4G recruitment (Roberts et al., 2015, 2017a), and a large bifurcated stem structure at base position nts 514-677 that bears the two auxiliary Y-1 and Y-2 tracts alongside and that appears to protrude out of the 5′ UTR. The Y-motifs are each positioned on bulges and are largely unpaired.

The “free-to-interact” potential of the auxiliary Y-motifs was experimentally tested using anti-sense single-stranded DNA oligonucleotides designed to block the CU-tracts accessibility, in virtue of their sequence complementarity (Figure 5B). To this end, we added DNA oligonucleotides consisting of the sequence complementarity to each of the Y-motifs up to 100 mM to the translation reaction of the full-length wild-type TriMV 1-739 RNA construct. As a control, we included anti-sense DNA oligonucleotides with complementary to the core Y_16_X_11_-AUG_740_ motif (anti-YX-AUG DNA), that we previously established to strongly and specifically interfere with TriMV IRES activity (Jaramillo-Mesa et al., 2019). We used unrelated human ß-globin oligo as a negative control (anti-B globin). Our result showed that, similarly to the effect of blocking the Y_16_X_11_-AUG_740_ motif for potential binding (Jaramillo-Mesa et al., 2019), translation steadily decreased in the presence of increasing fold molar excess of the anti-sense DNA oligonucleotides targeting each of the auxiliary Y motifs. These observations are in line with the importance of the accessibility of the Y-regions and the YX-AUG motifs to support translation.

### The CU-Rich Tracts Likely Increase the Cluster of Ribosomal Complexes to the 3′ End of the TriMV IRES

**Figure 6:**
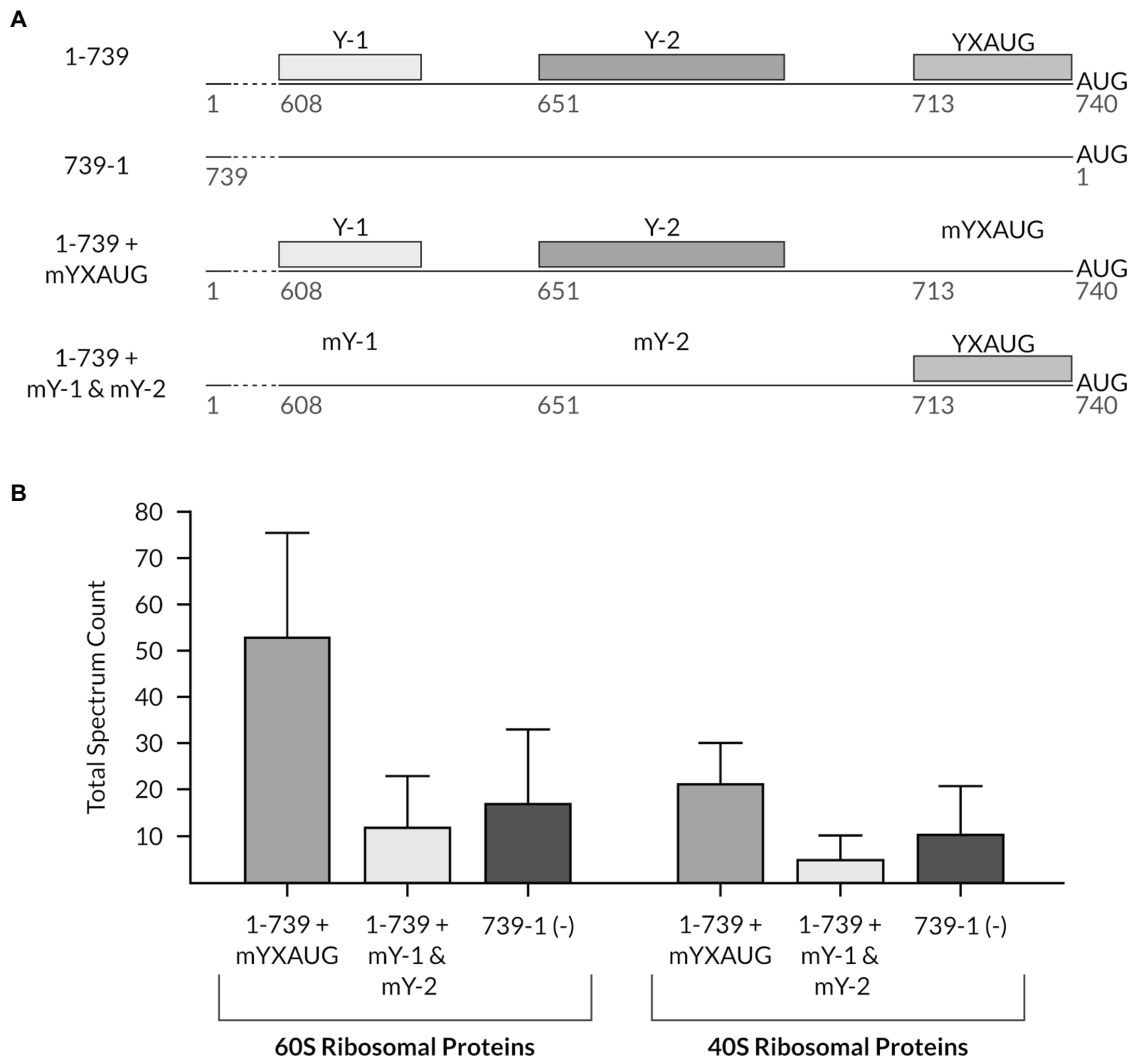
Mass spectrometry analysis reveals differential binding of translation initiation-related proteins on the TriMV 5′UTR mutants in the presence/absence of the multiple polypyrimidine tracts and the YX-AUG motif. **(A)** Schematic diagram of constructs tested in the experiment: 1-739 + mYXAUG in which the previously described YX-AUG motif (Jaramillo-Mesa et al., 2019) is replaced by 30 random Beta-globin nucleotides, but where both polypyrimidine regions (Y-1 and Y-2) are present; 1-739 + mY-1 & mY-2, where both polypyrimidine tracts are replaced with random nucleotides, but the YX-AUG motif is kept; and the non-functional TriMV reverse complementary sequence (739-1) used as a negative control. The wild-type 1-739 TriMV 5′UTR is shown for reference. **(B)** The total spectrum count of 40S and 60S ribosomal related proteins, which included the 60S ribosomal proteins L4, L3, L16, L2, L18e, L28e, P0, L14e, L18a, L13, L17, L15, L37, L19, and L23, and the 40S ribosomal proteins SA, S8, S3a, S4, S12, S25, S20, S6, and S26, was computed from the entire pool of identified proteins in the mass spectrometry analysis from two independent experiments and compared between the samples described in **(A)**. Detailed information of each protein can be found in the Supplementary Figure 3 and Supplementary Table 1. The mass spectrometry proteomics data have been deposited to the ProteomeXchange Consortium *via* the PRIDE (Perez-Riverol et al., 2022) partner repository and available with the dataset identifier PXD03144.

To next characterize the role of the auxiliary CU-rich tracts in ribosome recruitment on the TriMV IRES element, we set out to analyze the composition of the translation initiation complex specifically recruited by the Y-1 and Y-2 polypyrimidine tracks onto the viral 5′ UTR. To this end, we used (i) the TriMV 5′ UTR mutant in which the native YX-AUG motif was replaced with unrelated beta-globin sequence to maintain the same length of the 5′ UTR to that of a wild-type sequence. In this UTR construct, the Y1 and Y2 segments were intact (sample 1-739 + mYXAUG),; (ii) the mY-1+ mY-2 TriMV 5′ UTR mutant in which the native YX-AUG motif was intact but both Y1 and Y2 were mutated to random sequences (sample 1-739 + mY1 & mY2), and (iii) the reverse complementary sequence of the TriMV 5′ UTR (sample 739-1), as RNA baits for protein pull-down (Figure 6A). While each of those tested TriMV RNA sequences were functionally impaired for translation, our goal was to gain an insight on the pre-initiation complex that can be recruited by these RNA segments and their present motifs under stringent conditions. Each RNA segment bear at their 5′ end a stable hairpin structure to block 5′ entry of the ribosomes and to allow only internal recruitment of ribosomal complexes onto the RNA, and were biotinylated at their 3′ end. Native ribosomal complexes were let to assemble on those RNAs in the presence of non-hydrolyzable form of GTP, GMP-PNP, in the wheat germ translation reaction and next isolated and eluted through streptavidin beads. The GMP-PNP would arrest translation initiation at the formation of 48S ribosomal complex upon recognition of the preferred AUG and block the joining of the 60S large ribosomal subunit to the complex. The eluted complexes were next analyzed by mass spectrometry. This strategy would determine the enrichment of small and/or large ribosomal subunit proteins and their associated translation factors to each RNA. In particular, we set to compare any ribosomal complex enrichment with the TriMV 5′ UTR sequence in the presence or in the absence of the auxiliary CU-rich tracts (Figure 6B). We first confirmed that no translation was observed with our wild-type TriMV luciferase RNA construct in the presence of the GMP-PP (Supplementary Figure 2). The mass spectrometry analysis of two independent experiments identified a total of 455 host-interacting proteins clusters, including 142 uncharacterized proteins (Supplementary Figure 3 and Supplementary Table 1). We next addressed the composition of the translation initiation complex formed on the translationally impaired TriMV IRES elements that either missed the YX-AUG motif or the auxiliary CU-rich tracts (Figure 6B). The mass spec analysis indicated a strong enrichment of 60S ribosomal related proteins and 40S ribosomal related proteins in the eluate fraction from the mutant RNA that misses the YX-AUG motif but has intact CU-rich tracts (sample 1-739 + mYXAUG; Figure 6B) when compared to the fraction bound to the mutant that misses the CU-rich tracts but bears the YX-AUG motif, and the non-functional 739-1 RNA (sample 1-739 + mY-1 & m-Y2; Figure 6B). Additionally, some translation initiation factors mainly associated to the 40S ribosomal complex, including the eIF3 subunits, were predominantly recovered with the 1-739 + mYXAUG mutant (Supplementary Figure 3B and Supplementary Table 1). All of these observations are in line with a ribosome clustering function of the auxiliary CU-rich tracts.

### The Native YX-AUG Motif Is Preferentially Selected as the Start Site

**Figure 7:**
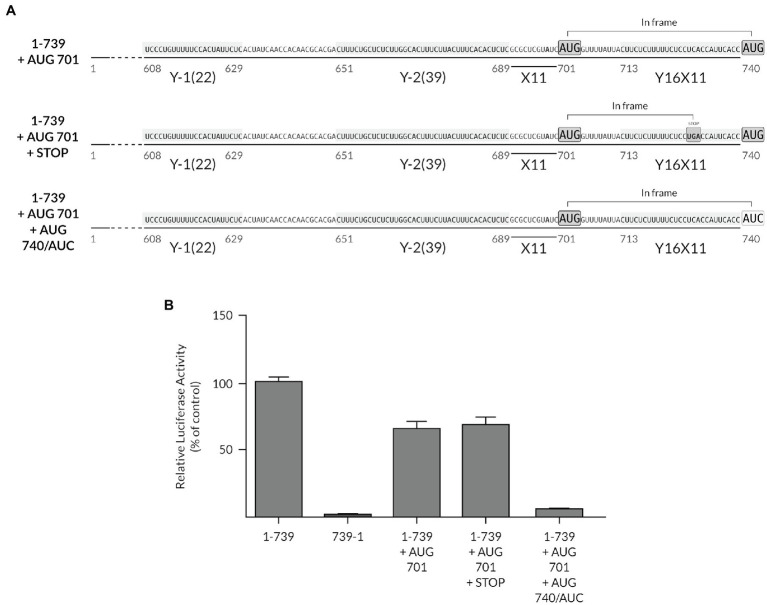
The native YX-AUG motif is preferentially selected as the start site in the presence of a proximal upstream AUG. **(A)** Schematic diagram of the 1-739 + AUG 701 mutants. Construct 1-739 + AUG 701 consists of the 1-739 TriMV leader with an AUG codon insertion at position 701, 11 nucleotides after the Y-2 motif, creating a Y_39_-X_11_-AUG_701_ motif upstream of the core original Y_16_X_11_-AUG motif. The inserted AUG codon is in-frame with the luciferase start codon AUG _740_. In the 1-739 + AUG 701 + STOP construct, an in-frame stop codon (UGA) is present between the two AUGs. In the 1-739 + AUG 701 + AUG 740/AUC construct, the luciferase start codon at position 740 is mutated to a non-start codon AUC. In all constructs, AUG _701_ shares the same optimal Kozak context as the wild-type AUG _740_ and is a good spacer length context from the closest CU-rich tract. **(B)** The relative luciferase activity in wheat germ extract of the TriMV 5′UTR mutants relativized to that of the wild-type sequence (construct 1-739). The non-functional TriMV reverse complementary sequence (739-1) is added as a negative control.

The potential role of the auxiliary Y-motifs in ribosome recruitment and their ability to functionally act as chimeric YX-AUG motifs led us to test whether we could re-designate the preferred start codon to upstream positions in the context of the full-length TriMV IRES. We thus inserted an uAUG at position 701 in the context of the full-length TriMV UTR and in frame with the downstream luciferase start codon (Figure 7A). In this context, we created a chimeric Y_39_X_11_-AUG_701_ motif followed by the original Y_16_X_11_-AUG_740_ motif that embeds the luciferase initiation site. Both AUGs are located 11 bases downstream of the closest polypyrimidine segments and in the same Kozak context. We hypothesized that the ribosomal complexes would linearly select the closest AUG codon embedded within the chimeric YX-AUG_701_ motif over the downstream native YX-AUG_740_ motif to initiate translation. We compared the *in vitro* translation efficiency of this mutant (construct 1-739 + AUG_701_) to that of the wild-type full-length TriMV 5′ UTR (construct 1-739) and the non-functional reverse complementary sequence (construct 739-1). Our result revealed that in the presence of the uAUG_701_ codon, translation of the luciferase was 70% of the wild-type level (construct 1-739 + AUG_701_). However, translation mainly occurred at the downstream native AUG_740_ codon. When the uAUG_701_ codon was followed by a stop codon, translation of the luciferase remained to similar level. Mutating the downstream AUG_740_ of the luciferase gene into an AUC codon abolished translation (Figure 7B). In sum, although the chimeric YX-AUG_701_ motif appears to be in good context for initiation, it was bypassed to favor preferential translation at the downstream native YX-AUG_740_ site, suggesting a non-linear mechanism of YX-AUG recognition and/or a better accessibility of the YX-AUG_740_ site in the context of the native UTR.

## Discussion

Diverse elements within the 5′ untranslated region of an mRNA can influence the translation efficiency at the main AUG codon, including secondary structures, modifications, and sequence motifs (Barrett et al., 2012). Here, we exposed the combinatory role of stretches of CU as cis-regulatory elements underlying the activity of the Triticum mosaic virus IRES. Polypyrimidine-rich tracts have been reported to contribute in the regulation of translation of different cellular and viral mRNAs. The 5′ terminal oligopyrimidine (5′TOP) motif, which is composed of 4–15 consecutive pyrimidines immediately downstream of the m7GpppG cap is essential for the regulation of the ribosomal protein encoding mRNAs (Levy et al., 1991). The class of TOP mRNAs are highly sensitive to translation repression in response to various stimuli through their recognition by the La-related protein 1 (LARP1) protein that binds to that 5′ end of the mRNA by blocking the assembly of the eIF4F complex (Fonseca et al., 2015; Lahr et al., 2017). Polypyrimidine tracts are also found in various IRESes (Lozano and Martinez-Salas, 2015). Similarly to our current finding for the TriMV IRES, multiple polypyrimidine tracts, in addition to the CU-rich segment within the picornavirus YX-AUG motif, have been mapped within different picornaviral IRES elements. In picornaviral IRESes, those tracts act as binding sites of the polypyrimidine tract binding protein (PTB) to stimulate translation (Romanelli et al., 2013). The consensus binding site of PTB are in general short RNA stretches such as UCUUC or UUCUC within a longer pyrimidine segment. The stringency of the PTB requirement in translation varies among the different picornaviral IRES elements from being essential for all type I IRESes, to stimulatory for the type II like EMCV (Kafasla et al., 2011). While its exact function in translation remains to be determined, the binding of the PTB to the IRES has been proposed to modulate the eIF4G binding on poliovirus IRES (Kafasla et al., 2010) or stabilize the structural fold of the EMCV IRES through its two binding sites, including the CU-tract within the picornavirus YX-AUG motif (Kafasla et al., 2009).

Plant virus IRES elements have also been reported to bear one to several polypyrimidine-rich tracts of various length. While we cannot dismiss that these polypyrimidine tracts similarly serve as a binding site for potential plant PTB homologous proteins, these segments have been proposed to be targets of the ribosomal complex through direct base pairing to a conserved region of the 18S rRNA. The 143-nt long uncapped *Tobacco etch virus* (family *Potyviridae*) 5′ leader, which is one of the initially well-characterized potyviral translation elements, contains two cap-independent regulatory elements (CIRE) of about 75 nucleotides long each that fold into pseudoknots (Zeenko and Gallie, 2005). The TEV CIREs contain two short oligopyrimidine stretches (nts 58–67 UUCUACUUCU and nts 84–94 UCAUUUCUUUU) that hybridize to the G-rich segment of the 18S rRNA. Disruption of the potential binding sites abolished translation activity of the TEV element (Zeenko and Gallie, 2005). Similar CU-rich regions complementary to the 18S rRNA have been reported within the 5′ leaders of several nepoviruses including *Blackcurrant reversion virus* (BRV, family *Comoviridae*; Karetnikov and Lehto, 2007, 2008). The 66-nt long BRV RNA 1 leader contains three 9–13 nt CU-rich segments with various complementarity to the 18S RNA. Deletion mutants that retain the 18S rRNA sequence complementarity on 2 out of the 3 segments retained about 50% of its translation efficiency. Reduction to only one binding site abolished IRES activity (Karetnikov and Lehto, 2008). Similarly the 161-nt long BRV RNA2 contains six CU-rich segments of 8–10 nts, and deletion of 2 out of 6 was sufficient to abolish translation (Karetnikov and Lehto, 2007). The stimulatory function of the polypyrimidine tract and its complementarity to the 18S rRNA was also reported for non-IRES-driven translation (Stupina et al., 2011). A single 19 nt-pyrimidine rich segment near the AUG codon of the 63 nt leader sequence of *Turnip crinkle virus* (TCV, Family *Tombusviridae*) RNA, was key for translation mediated by its 3′ cap-independent translation element (Stupina et al., 2011). Accumulating evidence supporting the function of multiple sites with 18S rRNA complementarity in translation are increasing (Deforges et al., 2015). The artificial introduction of two or more copies of complementarity sequences to region of the 18S rRNA to the 5′ leader of an uncapped mRNA considerably increased the binding to the 40S ribosomal subunit and cap-independent translation than a single copy (Akbergenov et al., 2004). However, none of those plant virus-related polypyrimidine tracts, except in TriMV as shown in our previous (Jaramillo-Mesa et al., 2019) and current studies, have been described in the context of a picornavirus-like YX-AUG motif. Our mass spectrometry analysis failed to identify any PTB-like proteins interacting with the TriMV IRES (Supplementary Table 1), in line with a different mode of action of the CU-rich tracts. However, it is worth noting that many of the interacting proteins identified in the mass spectrometry analysis have still uncharacterized functions in the wheat genome (Supplementary Table 1).

One key feature of IRES elements is that they are organized in multi-functional domains that work together as a single entity. In this study, we gained new insights on the modular domains of the TriMV IRES and their function. We have previously reported the 8-nt hairpin at position nts 469–488 that is crucial for eIF4G recruitment (Roberts et al., 2015, 2017a) and the 29-nts YX-AUG motif at position nts 720–739 for the recruitment of the ribosomal complex at the preferred initiation site (Stupina et al., 2011). Sequence analysis of the YX-AUG motif also revealed a potential direct contact of its CU-rich tract with the 18S RNA (Jaramillo-Mesa et al., 2019). While the YX-AUG-like motifs seems to be a conserved feature of the uncapped 5′ UTRs of virus members of the *Potyviridae* family with multiple AUGs, these 5′UTRs are unable to drive internal initiation (Jaramillo-Mesa et al., 2019). In fact, the inability of the TriMV YX-AUG motif alone to establish IRES activity on an unrelated plant viral 5′ UTR suggests that if the sequence complementary interaction is occurring, the potential contact to the 18S rRNA may not be involved in the initial recruitment of the ribosomal complexes to the mRNA, or that a recruitment/delivery mechanism may rely on multiple modular domains on the IRES element. One hypothesis is that the YX-AUG motif is involved in positioning the IRES−43S subunit in close proximity of the AUG codon to promote the 48S initiation complex, as proposed for the yeast histone 4 mRNA in which the complementarity to the 18S RNA near the AUG codon might be crucial for the proper position of the start codon at the entry site of the RNA channel (Martin et al., 2016). However, what is clear is that the contribution of the YX-AUG motif to translation initiation dependent on the viral strategy of translation is dictated by additional cis-acting regulatory elements on their 5′ UTRs.

Here, we uncover that the core YX-AUG motif function for TriMV IRES activity is highly dependent upon additional flanking polypyrimidine tracts. Our findings suggest that through a potential “bulk mechanism” approach, these motifs may help to cluster or increase the local concentration of the ribosomal complexes in the surrounding of the core YX-AUG and the correct initiation site and act as the initial recruitment site of the ribosomal complexes. First, we provided evidence that impaired translation due to the lack of the native YX-AUG motif can be reprogrammed by converting these auxiliary CU-rich tracts into chimeric YX-AUG-like motif by embedding an AUG codon to their close proximity. Secondly, this ability of the regions to redirect the assembly of translation machinery corroborated with the protein interactome data that indicated that in the presence of these motifs, the TriMV UTR sequence can interact with both the 40S and the 60S ribosomal subunits even in the absence of a functional YX-AUG motif and when the joining of the ribosomal complex was inhibited in the presence of non-hydrolyzable GTP. Thirdly, the “free-for-interaction” potential of these regions was validated by SHAPE analysis, in which both Y motifs are positioned on a long stem structure that appears to protrude out of the viral 5′ UTR. Furthermore, the high conservation of these regions among several newly reported TriMV isolates (Redila et al., 2021) suggests a selective pressure to suppress any sequence changes around the overall region and is in support of the essential role of these polypyrimidine motifs.

One of the novelties in this study is that we uncovered a new layer of control of the selection of the initiation site, that has not been extensively studied, especially in plant-related mRNAs. The translation of an mRNA depends heavily on the ability to initiate at the correct initiation site. Leaky scanning mechanism selects for an optimal Kozak sequence context of an AUG codon (Kozak, 1999). In contrast to leaky scanning, sliding of the ribosomal complex to the next AUG can be mechanically triggered by a delay in the eIF2-bound GTP hydrolysis, which is controlled by the eukaryotic translation initiation factor eIF5 (Loughran et al., 2012; Terenin et al., 2016). eIF5 promotes the hydrolysis of eIF2-bound GTP and the release of the Pi in response to the initiation codon recognition and irreversibly stabilizes the 60S subunit joining for translation to initiate (Paulin et al., 2001). A delay in the eIF5 activity results in the sliding of the ribosomal complex to the next AUG to increase the stringency of the start codon selection (Loughran et al., 2012; Terenin et al., 2016). Here, we provided evidence that the initial distance between the landing site of the ribosomal complex and the first encountered AUG may contribute in the selection process of the favorable initiation site. Our results with the chimeric YX-AUG motif showed that a shorter distance, within a 11-nt range, between the CU-rich tract and the embedded AUG codon within the YX-AUG motif favors commitment of the ribosomal complex to initiate translation at the first encountered AUG codon. However, a longer distance, starting at 18 bases, was sufficient to lead to the slippage of the ribosomal complex to the next downstream AUG codon, likely through ribosomal scanning. Whatever the mechanism may be by which the ribosomes skip the first AUG, it is clear that that the ribosome complexes are capable of scanning long distance to reach the next AUG codon. Intriguingly, the chimeric YX-AUG motif failed to reprogram translation in the context of the full-length UTR. The data suggest that the recruited ribosomal complexes preferentially reach and initiate at the native unstructured downstream YX-AUG motif and thus bypass in a non-linear fashion the segment of the long stem structure that embeds the chimeric YX-AUG motif. Additional works are needed to link ribosome processivity and the role of the secondary structures that could block accessibility of the first AUG as shown with the human rhinovirus 3 IRES (Kaminski et al., 2010).

In conclusion, TriMV dedicates at least 7% of its genome, which corresponds to its entire 739-nts 5′ UTR, to internally recruit the ribosomes and drive translation. An emerging view of the function of the modular domains of the TriMV IRES is that upstream regions of the TriMV leader sequence, including the auxiliary CU-rich tracts located on a long protruding stem structure direct the initial clustering of the ribosomal complexes to the 3′ end of the IRES element. With the adjacent hairpin that delivers the scaffold eIF4G protein, it coordinates the formation of the 48S complex on the correct AUG embedded within the YX-AUG motif located downstream of the stem structure. This proposed model provides a platform to generate new questions such as: how do the modular domains of the TriMV IRES orchestrate each step of translation initiation? During the viral life cycle, does the TriMV IRES assume alternative (active vs. inactive) RNA configurations to timely and spatially control its protein expression? In addition to the translation initiation factors eIF4G and eIF4A that were previously identified as essential for TriMV IRES activity (Roberts et al., 2015, 2017a) what are the other cellular RNA binding proteins that coordinate the recruitment and assembly of the translation machinery on the mRNA? Although many questions arise and further work is needed, the information provided by this study paves the way to better understand the core requirements and potentially conserved cap-independent translation mechanisms in plant hosted RNA viruses.

## Data Availability Statement

The original contributions presented in the study are publicly available. The mass spectrometry data can be found at ProteomeXchange Consortium via the PRIDE (Perez-Riverol et al., 2022) partner repository and available with the dataset identifier PXD031443.

## Author Contributions

AR and HJ-M conceived and coordinated the study, interpreted the data, and wrote the manuscript. HJ-M performed all experiments. EF contributed in the SHAPE assay. All authors contributed to the article and approved the submitted version.

## Funding

This work was supported by the Hatch Act Formula Fund (MSN169250) and a Wisconsin Alumni Research Foundation Fund (MSN194345) to AR.

## Conflict of Interest

The authors declare that the research was conducted in the absence of any commercial or financial relationships that could be construed as a potential conflict of interest.

## Publisher’s Note

All claims expressed in this article are solely those of the authors and do not necessarily represent those of their affiliated organizations, or those of the publisher, the editors and the reviewers. Any product that may be evaluated in this article, or claim that may be made by its manufacturer, is not guaranteed or endorsed by the publisher.
