# Supplementary material for: Multiple Cis-acting Polypyrimidine Tract Elements Regulate a Cooperative Mechanism for Triticum Mosaic Virus Internal Ribosomal Entry Site Activity
Source: Front Plant Sci. 2022 Apr 12;13:864832. doi: 10.3389/fpls.2022.864832 (PMC9042117; doi:10.3389/fpls.2022.864832)
Supplement: Supplementary file 1 [file Data_Sheet_1.PDF]

Supplementary Material

1.1 Supplementary Figures

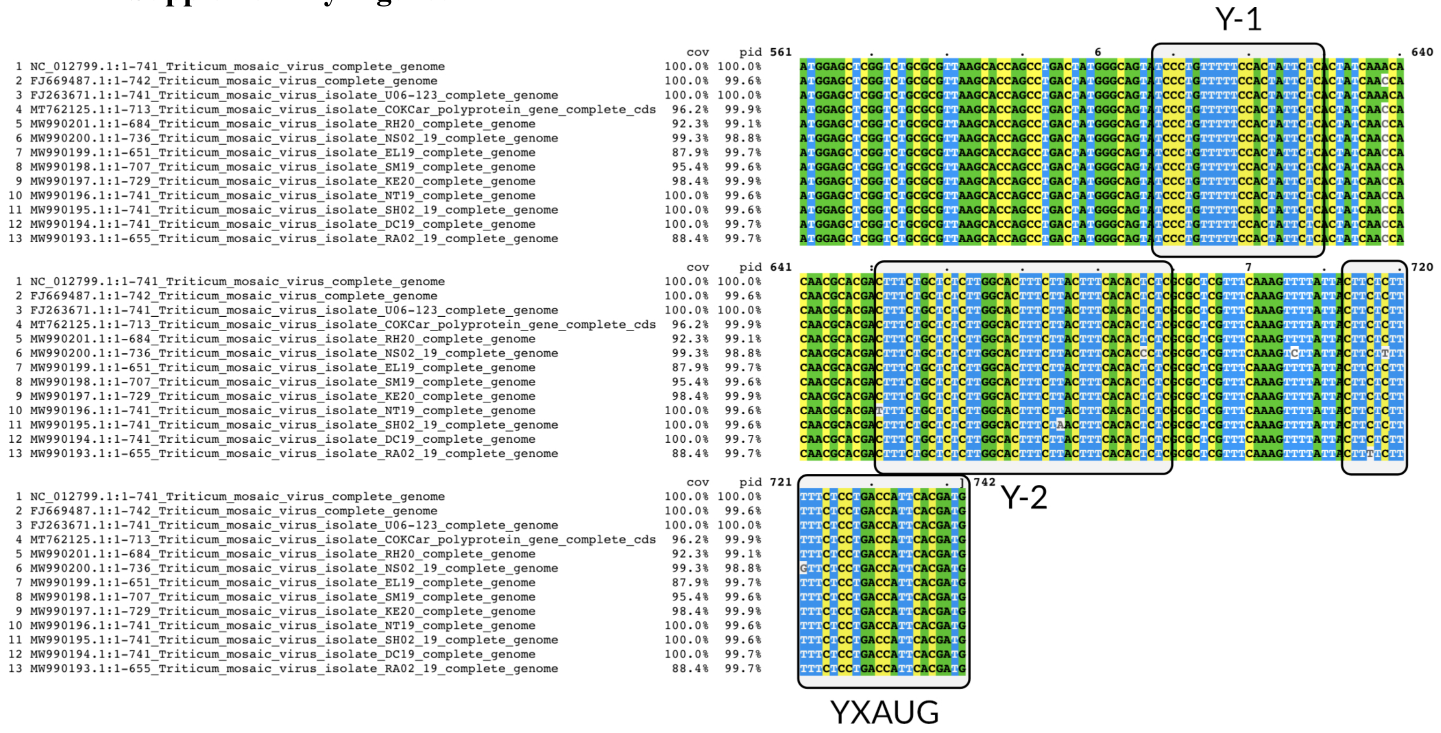

Supplementary Figure 1. TriMV isolates exhibit high conservation of the multiple polypyrimidine tracts present in the 5'UTR region. Multiple sequence alignment of the 5'UTR region 561-742 of 13 TriMV isolates. Y-1 (position 608 – 629), Y2 (position 651 – 689) polypyrimidine tracts and the previously described YX-AUG motif (positions 713-740) are highlighted in black boxes.

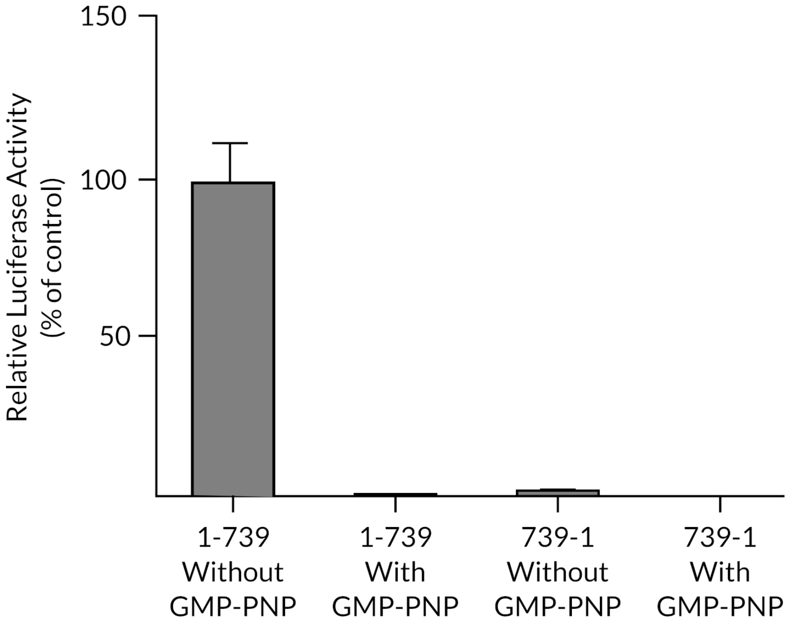

Supplementary Figure 2. Addition of GMP-PNP *in vitro* inhibits translation. The relative luciferase activity in wheat germ extract of the wildtype TriMV 5'UTR (1-739) and the nonfunctional TriMV reverse complementary sequence (739-1) in the presence or absence of the non-hydrolysable GTP analog, GMP-PNP, which prevents 80S ribosomal complex formation and therefore, translation initiation. The relative luciferase activity in wheat germ extract of the different sample is relativized to that of the TriMV wild-type sequence (1-739 without GMP-PNP).

A

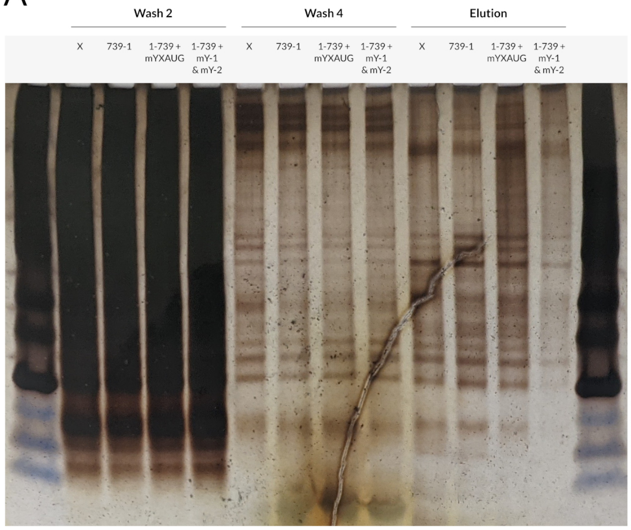

B

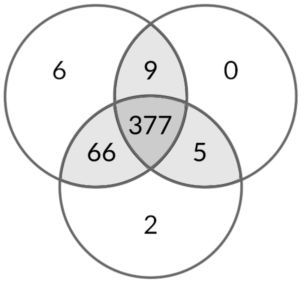

C

Identified Proteins	Organism	Accession Number	Molecular Weight	Average Spectral Count		
				1-739 + mYXAUG	1-739 + mY-1 & mY-2	739-1
**60S Ribosomal proteins**						
Cluster of Ribos_L4_asso_C domain-containing protein	*Triticum aestivum*	A0A3B5YZJ4	44 kDa	11	2	5
Cluster of Ribosomal protein L3	*Triticum aestivum*	Q6V959	45 kDa	9	2	2
Ribosomal_L16 domain-containing protein	*Triticum aestivum*	A0A0C4BIR6	25 kDa	5	1	2
60S acidic ribosomal protein P0	*Triticum aestivum*	A0A3B6RK63	35 kDa	4	2	1
Ribosomal_L2_C domain-containing protein	*Triticum aestivum*	W5ECL2	28 kDa	4	1	1
Ribosomal_L18e/L15P domain-containing protein	*Triticum aestivum*	A0A341P0J7	21 kDa	3	2	0
Cluster of Ribosomal_L28e domain-containing protein	*Triticum aestivum*	A0A3B5Z336	17 kDa	3	1	1
Ribosomal_L14e domain-containing protein	*Triticum aestivum*	A0A096ULG3	15 kDa	3	0	1
Cluster of 60S ribosomal protein L18a	*Triticum aestivum*	A0A1D5SRX8	21 kDa	2	1	2
Cluster of 60S ribosomal protein L13	*Triticum aestivum*	A0A3B6RJP2	24 kDa	2	1	1
Ribosomal protein L17	*Triticum aestivum*	Q5I7K4	15 kDa	2	1	2
Ribosomal protein L15	*Triticum aestivum*	A0A1D5UYA3	22 kDa	2	0	1
Ribosomal protein L37	*Triticum aestivum*	A0A0C4BK42	11 kDa	2	1	1
Ribosomal_L18e/L15P domain-containing protein	*Triticum aestivum*	A0A3B6ATQ1	21 kDa	1	1	0
Cluster of Ribosomal protein L19	*Triticum aestivum*	A0A1D5TPB6	24 kDa	1	0	0
Ribosomal_L18_c domain-containing protein	*Triticum aestivum*	A0A3B6LRC5	35 kDa	1	0	1
Ribosomal_L23eN domain-containing protein	*Triticum aestivum*	A0A3B6B204	20 kDa	1	0	0
**40S Ribosomal proteins**						
Cluster of 40S ribosomal protein SA	*Triticum aestivum*	A0A3B6HQ62	32 kDa	6	2	4
40S ribosomal protein S8	*Triticum aestivum*	A0A1D5UKZ4	25 kDa	3	0	2
40S ribosomal protein S3a	*Triticum aestivum*	A0A3B6IU74	30 kDa	3	0	0
40S ribosomal protein S4	*Triticum aestivum*	A0A1D5SLI4	30 kDa	2	1	1
40S ribosomal protein S12	*Triticum aestivum*	W5FEZ3	15 kDa	2	0	2
40S ribosomal protein S25	*Triticum aestivum*	A0A3B6HY18	11 kDa	2	1	1
Ribosomal protein S20	*Triticum aestivum*	U5HTD8	14 kDa	2	1	1
Cluster of 40S ribosomal protein S6	*Triticum aestivum*	W5C8N6	28 kDa	1	1	1
40S ribosomal protein S26	*Triticum aestivum*	A0A3B6KQB8	15 kDa	1	0	1
Ribosomal_S7 domain-containing protein	*Triticum aestivum*	A0A3B6EC38	22 kDa	1	1	1
**Translation Factors**						
Elongation factor 1-alpha	*Triticum aestivum*	A0A1D5XDB6	49 kDa	12	5	9
Eukaryotic translation initiation factor 3 subunit B	*Triticum aestivum*	A0A3B6LV25	83 kDa	6	3	2
Eukaryotic translation initiation factor 5B	*Triticum aestivum*	A0A3B5Z6Z8	143 kDa	5	4	0
Eukaryotic translation initiation factor 3 subunit I	*Triticum aestivum*	A0A3B6HW61	38 kDa	3	2	3
Eukaryotic translation initiation factor 3 subunit C	*Triticum aestivum*	A0A3B6KJI3	105 kDa	5	1	0
Eukaryotic translation initiation factor 3 subunit A	*Triticum aestivum*	A0A3B6GJX5	115 kDa	4	0	0
Eukaryotic translation initiation factor 3 subunit G	*Triticum aestivum*	A0A3B6NWB4	31 kDa	2	1	1
Eukaryotic translation initiation factor 3 subunit L	*Triticum aestivum*	A0A3B6EFG4	61 kDa	1	2	1
Eukaryotic translation initiation factor 3 subunit H	*Triticum aestivum*	A0A3B6C6H6	38 kDa	2	2	1
Eukaryotic translation initiation factor 3 subunit D	*Triticum aestivum*	A0A3B5Y6B4	66 kDa	1	1	1
Eukaryotic translation initiation factor 3 subunit F	*Triticum aestivum*	A0A3B6NM42	32 kDa	1	1	0
Eukaryotic translation initiation factor 3 subunit M	*Triticum aestivum*	A0A3B6AQL4	46 kDa	1	0	0
Eukaryotic translation initiation factor 3 subunit E	*Triticum aestivum*	A0A3B6KK98	51 kDa	1	1	0

Supplementary Figure 3. Mass spectrometry analysis reveals differential binding of translation initiation-related proteins for TriMV 5'UTR mutants in the presence/absence of multiple polypyrimidine tracts and the YX-AUG motif. The following constructs were used in the experiment: 1-739 + mYXAUG in which the YX-AUG motif is replaced by 30 random Beta globin nucletoides, but where both polypyrimidine regions (Y-1 and Y-2) are present; 1-739 + mY-1 & mY-2, where both polypyrimidine tracts are replaced with random nucleotides, but the YXAUG is kept; and the nonfunctional TriMV reverse complementary sequence (739-1) used as a negative control. **A)** A representative silver-stained SDS-PAGE gel of the wash and elution fractions from the pull-down assays for each tested sample. Lane 1 corresponded to the protein ladder (BioRad 10–250 kD Precision Plus Protein Dual Color Standard). Lane 2-5 corresponded to the 2nd wash fractions from each sample. Lanes 6-9 corresponded to the 4th wash fractions from each sample. Lane 10-13 corresponded to the final elution for each sample. The sample corresponding to each of the lanes is labeled accordingly in the figure. Lanes labeled as X were not included in the mass spectrometry analysis. A 10ul aliquot was loaded in each corresponding sample. **B)** Total spectrum count of protein clusters identified in the mass spectrometry analysis. A total of 455 protein clusters were identified, including 142 uncharacterized proteins (PRIDE accession PXD031443). The figure was generated using the Scaffold software (Proteome Software, Inc). **C)** List of the components of the translation machinery that were identified from the mass spectrometry data and compared to the *Triticum aestivum* UniProt reference proteome. The accession numbers, molecular weight and total spectrum count for each sample in the two experimental replicates are indicated in the table. Raw data can be accessed in the PRIDE database accession PXD031443 and in Supplementary Table 1.

Supplementary Table 1. Mass spectrometry data analysis of the identified protein factors that interact with the RNA samples 739-1, 1-739-mYXAUG and 1-739-mY1mY2 in two independent replicates. The data was run against the *Triticum aestivum* (130,673 entries) UniProt reference proteome database (Proteome ID UP000019116) and results were analyzed using the Scaffold software. Total spectrum counts for each sample are shown. Raw data is available at PRIDE repository with the identifier PXD031443.

			Total Spectrum Count					
**Identified Proteins**	**Accession Number**	**Molecular Weight**	**739-1 (1)**	**739-1 (2)**	**1-739-mYXAUG (1)**	**1-739-mYXAUG (2)**	**1-739-mY1mY2 (1)**	**1-739-mY1mY2 (2)**
Cluster of Uncharacterized protein OS=Triticum aestivum OX=4565 PE=4 SV=1 (A0A3B5Z3G6)	A0A3B5Z3G6 [3]	74 kDa	40	118	76	104	17	79
Cluster of Uncharacterized protein OS=Triticum aestivum OX=4565 PE=3 SV=1 (A0A3B6HPZ5)	A0A3B6HPZ5 [15]	72 kDa	17	104	27	93	7	54
Cluster of Uncharacterized protein OS=Triticum aestivum OX=4565 PE=4 SV=1 (A0A3B5YTY7)	A0A3B5YTY7 [2]	38 kDa	12	50	23	43	2	29
Cluster of Uncharacterized protein OS=Triticum aestivum OX=4565 PE=3 SV=1 (A0A3B6ISW5)	A0A3B6ISW5 [3]	39 kDa	23	44	18	56	0	39
Em protein OS=Triticum aestivum OX=4565 GN=EM PE=2 SV=1	P04568	10 kDa	13	28	16	31	8	34
Cluster of Uncharacterized protein OS=Triticum aestivum OX=4565 PE=3 SV=1 (W4ZP51)	W4ZP51 [2]	10 kDa	15	35	15	33	7	32
Cluster of Ricin B-type lectin domain-containing protein OS=Triticum aestivum OX=4565 PE=4 SV=1 (A0A3B6HVK5)	A0A3B6HVK5 [3]	39 kDa	11	32	10	16	9	5
Cluster of Uncharacterized protein OS=Triticum aestivum OX=4565 PE=4 SV=1 (A0A3B5XYS8)	A0A3B5XYS8 [5]	62 kDa	7	35	11	24	0	26
Cluster of Aldo_ket_red domain-containing protein OS=Triticum aestivum OX=4565 PE=3 SV=1 (A0A3B5ZYX1)	A0A3B5ZYX1 [2]	36 kDa	4	39	9	36	1	16
Cluster of Tr-type G domain-containing protein OS=Triticum aestivum OX=4565 PE=3 SV=1 (A0A3B6RQD1)	A0A3B6RQD1 [5]	94 kDa	2	34	8	27	1	10
Cluster of Glyceraldehyde-3-phosphate dehydrogenase OS=Triticum aestivum OX=4565 PE=3 SV=1 (A0A3B6RKE1)	A0A3B6RKE1 [5]	36 kDa	2	28	8	26	2	17
Cluster of MBD domain-containing protein OS=Triticum aestivum OX=4565 PE=4 SV=1 (A0A3B6MXQ8)	A0A3B6MXQ8 [5]	50 kDa	7	10	5	9	2	23
Cluster of Uncharacterized protein OS=Triticum aestivum OX=4565 PE=4 SV=1 (A0A3B6FP91)	A0A3B6FP91 [2]	43 kDa	7	23	8	22	0	19
Cluster of Uncharacterized protein OS=Triticum aestivum OX=4565 PE=4 SV=1 (A0A3B6LUV8)	A0A3B6LUV8 [2]	57 kDa	3	25	7	22	5	5
Cluster of Peroxiredoxin OS=Triticum aestivum OX=4565 GN=CAMPLR22A2D_LOCUS5300 PE=2 SV=1 (D0PRB4)	D0PRB4 [2]	24 kDa	5	27	6	28	1	13
Cluster of SHSP domain-containing protein OS=Triticum aestivum OX=4565 PE=3 SV=1 (A0A3B6SG07)	A0A3B6SG07 [2]	18 kDa	4	18	7	13	3	8
Cluster of Elongation factor 1-alpha OS=Triticum aestivum OX=4565 PE=3 SV=1 (A0A1D5XDB6)	A0A1D5XDB6 [4]	49 kDa	1	17	5	18	2	7
Cluster of Heat shock protein 90 OS=Triticum aestivum OX=4565 GN=HSP90-2 PE=1 SV=1 (Q0Q0I7)	Q0Q0I7 [2]	80 kDa	2	22	8	25	0	7
Cluster of Phosphoglycerate kinase OS=Triticum aestivum OX=4565 PE=3 SV=1 (A0A3B6NNE0)	A0A3B6NNE0 [2]	42 kDa	7	22	7	21	1	9
Cluster of Phosphopyruvate hydratase OS=Triticum aestivum OX=4565 PE=3 SV=1 (A0A3B6KAJ3)	A0A3B6KAJ3 [5]	48 kDa	9	28	9	26	0	4
Cluster of Fructose-bisphosphate aldolase OS=Triticum aestivum OX=4565 GN=FBA16 PE=3 SV=1 (A0A2P1E8S5)	A0A2P1E8S5 [3]	39 kDa	1	28	6	26	0	8
Cluster of Uncharacterized protein OS=Triticum aestivum OX=4565 PE=3 SV=1 (A0A3B6HRX1)	A0A3B6HRX1 [2]	27 kDa	7	12	9	17	2	12
Cluster of Uncharacterized protein OS=Triticum aestivum OX=4565 PE=4 SV=1 (A0A3B5ZRE8)	A0A3B5ZRE8 [5]	93 kDa	2	25	3	25	0	8
Cluster of Uncharacterized protein OS=Triticum aestivum OX=4565 PE=3 SV=1 (A0A3B5ZWI4)	A0A3B5ZWI4 [3]	17 kDa	5	16	5	18	0	20
Cluster of Uncharacterized protein OS=Triticum aestivum OX=4565 PE=4 SV=1 (A0A3B6TRJ3)	A0A3B6TRJ3 [3]	59 kDa	2	1	9	16	1	10
Uncharacterized protein OS=Triticum aestivum OX=4565 PE=3 SV=1	A0A3B6EFK5	42 kDa	6	17	5	17	0	12
Uncharacterized protein OS=Triticum aestivum OX=4565 PE=4 SV=1	A0A3B6KNF4	56 kDa	2	16	6	12	5	6
Cluster of Uncharacterized protein OS=Triticum aestivum OX=4565 PE=3 SV=1 (A0A3B6JL62)	A0A3B6JL62 [2]	30 kDa	5	16	8	14	2	13
Cluster of Peptidyl-prolyl cis-trans isomerase OS=Triticum aestivum OX=4565 GN=CyP3 PE=1 SV=1 (Q93W25)	Q93W25 [2]	18 kDa	5	18	5	21	1	5
Cluster of Alcohol dehydrogenase ADH1A OS=Triticum aestivum OX=4565 PE=2 SV=1 (A9U8G4)	A9U8G4 [2]	41 kDa	2	22	6	19	0	13
Cluster of Adenosylhomocysteinase OS=Triticum aestivum OX=4565 GN=SAHH PE=2 SV=1 (P32112)	P32112 [2]	53 kDa	4	19	3	13	0	11
Cluster of UTP--glucose-1-phosphate uridylyltransferase OS=Triticum aestivum OX=4565 PE=3 SV=1 (A0A1D6S518)	A0A1D6S518 [3]	52 kDa	4	19	3	20	0	9
Cluster of SHSP domain-containing protein OS=Triticum aestivum OX=4565 PE=3 SV=1 (A0A3B6IQX6)	A0A3B6IQX6 [2]	17 kDa	6	11	6	9	4	5
Cluster of Lysine--tRNA ligase OS=Triticum aestivum OX=4565 PE=3 SV=1 (A0A3B6KRW5)	A0A3B6KRW5 [3]	68 kDa	1	14	2	14	2	6
Uncharacterized protein OS=Triticum aestivum OX=4565 PE=3 SV=1	A0A3B5ZUU2	10 kDa	7	11	10	9	3	12
Cluster of Heat shock protein 90 OS=Triticum aestivum OX=4565 PE=3 SV=1 (A0A3B6SIR8)	A0A3B6SIR8 [3]	74 kDa	2	23	7	18	0	3
Cluster of Uncharacterized protein OS=Triticum aestivum OX=4565 GN=TRAES_3BF023000010CFD_c1 PE=3 SV=1 (W5CXB0)	W5CXB0	73 kDa	3	18	7	17	0	7
Cluster of Uncharacterized protein OS=Triticum aestivum OX=4565 PE=4 SV=1 (A0A3B6CCY6)	A0A3B6CCY6 [2]	76 kDa	0	0	9	16	0	7
Cluster of Protein disulfide-isomerase OS=Triticum aestivum OX=4565 GN=PDI3 PE=2 SV=1 (Q93XQ7)	Q93XQ7 [6]	57 kDa	2	14	6	16	0	8
Cluster of Uncharacterized protein OS=Triticum aestivum OX=4565 PE=3 SV=1 (A0A3B6PJY5)	A0A3B6PJY5	62 kDa	2	13	4	20	0	7
Cluster of Uncharacterized protein OS=Triticum aestivum OX=4565 PE=3 SV=1 (A0A3B6KMM5)	A0A3B6KMM5 [2]	27 kDa	6	15	5	16	2	8
Cluster of Phosphoglucomutase (alpha-D-glucose-1,6-bisphosphate-dependent) OS=Triticum aestivum OX=4565 PE=3 SV=1 (A0A3B6IK29)	A0A3B6IK29 [2]	70 kDa	6	14	3	17	0	11
Malate dehydrogenase OS=Triticum aestivum OX=4565 PE=3 SV=1	A0A3B5XY18 (+2)	35 kDa	4	14	9	15	1	9
Heat shock protein 90 OS=Triticum aestivum OX=4565 GN=Hsp90.1-A1 PE=2 SV=1	F4Y589 (+1)	81 kDa	2	18	5	17	0	4
Cluster of Uncharacterized protein OS=Triticum aestivum OX=4565 PE=3 SV=1 (A0A3B6EEA7)	A0A3B6EEA7 [3]	27 kDa	6	13	5	12	0	9
Cluster of Poly [ADP-ribose] polymerase OS=Triticum aestivum OX=4565 PE=3 SV=1 (A0A3B5Z1F3)	A0A3B5Z1F3 [3]	92 kDa	0	20	5	18	0	7
Cluster of Uncharacterized protein OS=Triticum aestivum OX=4565 PE=3 SV=1 (A0A3B6KDW0)	A0A3B6KDW0 [7]	45 kDa	2	9	2	13	1	4
Cluster of Uncharacterized protein OS=Triticum aestivum OX=4565 PE=3 SV=1 (A0A3B5ZZP8)	A0A3B5ZZP8 [5]	23 kDa	5	13	7	8	1	13
Cluster of SHSP domain-containing protein OS=Triticum aestivum OX=4565 PE=3 SV=1 (A0A1D5WW00)	A0A1D5WW00 [4]	17 kDa	4	13	5	9	2	3
Cluster of Ribos_L4_asso_C domain-containing protein OS=Triticum aestivum OX=4565 PE=3 SV=1 (A0A3B5YZJ4)	A0A3B5YZJ4 [5]	44 kDa	0	10	6	16	0	4
Cluster of Lipoxygenase OS=Triticum aestivum OX=4565 PE=3 SV=1 (A0A3B6LRK8)	A0A3B6LRK8 [5]	97 kDa	0	21	2	12	0	5
Cluster of Uncharacterized protein OS=Triticum aestivum OX=4565 PE=3 SV=1 (A0A3B6SF94)	A0A3B6SF94 [2]	28 kDa	4	12	7	11	1	6
Cluster of Aspartate--tRNA ligase OS=Triticum aestivum OX=4565 PE=3 SV=1 (A0A3B6KGM6)	A0A3B6KGM6 [3]	61 kDa	3	8	0	12	1	5
Cluster of Uncharacterized protein OS=Triticum aestivum OX=4565 PE=3 SV=1 (A0A3B6GTM0)	A0A3B6GTM0 [2]	28 kDa	2	20	4	11	0	6
Uncharacterized protein OS=Triticum aestivum OX=4565 PE=3 SV=1	A0A3B6NJT9 (+1)	73 kDa	2	13	2	14	1	8
Polyadenylate-binding protein OS=Triticum aestivum OX=4565 PE=3 SV=1	A0A3B6LKB2 (+1)	71 kDa	4	2	6	8	5	3
Uncharacterized protein OS=Triticum aestivum OX=4565 PE=4 SV=1	A0A3B6RM24 (+1)	47 kDa	0	10	6	11	0	4
Cluster of Uncharacterized protein OS=Triticum aestivum OX=4565 PE=3 SV=1 (A0A3B5YV41)	A0A3B5YV41 [2]	61 kDa	0	13	1	12	0	5
Cluster of Uncharacterized protein OS=Triticum aestivum OX=4565 PE=3 SV=1 (A0A3B6JLR9)	A0A3B6JLR9 [3]	17 kDa	4	10	5	5	1	1
Cluster of Uncharacterized protein OS=Triticum aestivum OX=4565 PE=4 SV=1 (A0A3B6NY09)	A0A3B6NY09	34 kDa	4	4	3	9	4	7
Cluster of Ribosomal protein L3 OS=Triticum aestivum OX=4565 GN=RPL3 PE=2 SV=1 (Q6V959)	Q6V959 [4]	45 kDa	0	3	5	12	0	3
Cluster of VOC domain-containing protein OS=Triticum aestivum OX=4565 PE=4 SV=1 (A0A3B6IS82)	A0A3B6IS82 [2]	15 kDa	3	9	4	7	1	7
Cluster of Prolyl-tRNA synthetase OS=Triticum aestivum OX=4565 PE=3 SV=1 (A0A3B5Z1S9)	A0A3B5Z1S9 [3]	57 kDa	3	13	3	9	3	2
Cluster of Ribonuclease OS=Triticum aestivum OX=4565 PE=4 SV=1 (A0A3B6JCE1)	A0A3B6JCE1 [2]	109 kDa	0	3	5	14	0	4
Pyruvate decarboxylase OS=Triticum aestivum OX=4565 PE=3 SV=1	A0A3B6JJ66	65 kDa	1	15	3	12	0	5
VOC domain-containing protein OS=Triticum aestivum OX=4565 PE=4 SV=1	A0A3B6JIQ3	15 kDa	3	6	4	10	1	7

Cluster of Beta-amylase OS=Triticum aestivum OX=4565 PE=3 SV=1 (A0A3B6KSH4)	A0A3B6KSH4 [7]	59 kDa	1	15	2	11	0	3
Cluster of Clp R domain-containing protein OS=Triticum aestivum OX=4565 PE=3 SV=1 (A0A3B5Z1T0)	A0A3B5Z1T0 [5]	101 kDa	0	14	5	14	0	4
Cluster of Uncharacterized protein OS=Triticum aestivum OX=4565 PE=4 SV=1 (A0A3B6JLF7)	A0A3B6JLF7 [2]	46 kDa	4	5	3	5	1	11
Cluster of ATP synthase subunit beta OS=Triticum aestivum OX=4565 PE=3 SV=1 (A0A3B6EIN4)	A0A3B6EIN4 [4]	59 kDa	1	9	0	10	0	4
Cluster of 5-methyltetrahydropteroyltriglutamate--homocysteine S-methyltransferase OS=Triticum aestivum OX=4565 PE=3 SV=1 (A0A3B6K9Q1)	A0A3B6K9Q1 [9]	89 kDa	0	11	2	16	0	4
Cluster of Uncharacterized protein OS=Triticum aestivum OX=4565 PE=4 SV=1 (A0A3B6HMU5)	A0A3B6HMU5 [4]	91 kDa	0	12	4	14	0	3
Cluster of W2 domain-containing protein OS=Triticum aestivum OX=4565 PE=3 SV=1 (A0A3B6HUI5)	A0A3B6HUI5 [2]	47 kDa	0	6	3	10	0	2
Cluster of Uncharacterized protein OS=Triticum aestivum OX=4565 PE=3 SV=1 (A0A3B6RB14)	A0A3B6RB14 [4]	64 kDa	2	11	1	10	0	3
Glyoxalase I OS=Triticum aestivum OX=4565 PE=3 SV=1	A0A1D6CB88 (+2)	33 kDa	2	13	2	11	0	1
Cluster of ATP synthase subunit alpha OS=Triticum aestivum OX=4565 PE=3 SV=1 (A0A3B6JHJ0)	A0A3B6JHJ0 [3]	56 kDa	2	6	4	4	0	1
Cluster of Uncharacterized protein OS=Triticum aestivum OX=4565 PE=4 SV=1 (A0A3B5ZVX4)	A0A3B5ZVX4 [2]	18 kDa	3	7	6	6	0	8
Cluster of Uncharacterized protein OS=Triticum aestivum OX=4565 PE=3 SV=1 (A0A3B6KKC0)	A0A3B6KKC0 [2]	26 kDa	1	13	4	11	0	1
Cluster of Peroxidase OS=Triticum aestivum OX=4565 GN=TRAES_3BF072300070CFD_c1 PE=3 SV=1 (A0A077S7C3)	A0A077S7C3 [2]	39 kDa	3	7	2	14	0	5
Cluster of MI domain-containing protein OS=Triticum aestivum OX=4565 PE=4 SV=1 (A0A3B6C954)	A0A3B6C954 [3]	87 kDa	1	4	3	4	1	3
Cluster of Malic enzyme OS=Triticum aestivum OX=4565 PE=3 SV=1 (A0A3B6GYC1)	A0A3B6GYC1	76 kDa	2	14	3	10	0	4
Nucleoside diphosphate kinase OS=Triticum aestivum OX=4565 PE=3 SV=1	A0A3B5Y072 (+2)	17 kDa	3	8	3	8	1	3
Cluster of Adenosine kinase OS=Triticum aestivum OX=4565 PE=3 SV=1 (A0A3B6NQW1)	A0A3B6NQW1 [3]	37 kDa	3	8	5	10	0	4
Cluster of Uncharacterized protein OS=Triticum aestivum OX=4565 PE=4 SV=1 (A0A3B6NLU0)	A0A3B6NLU0 [3]	37 kDa	0	9	3	8	0	4
Uncharacterized protein OS=Triticum aestivum OX=4565 PE=4 SV=1	A0A3B6NS49 (+3)	65 kDa	2	9	1	12	0	3
Cluster of Phosphoenolpyruvate carboxylase OS=Triticum aestivum OX=4565 PE=3 SV=1 (A0A3B6KHJ0)	A0A3B6KHJ0 [5]	110 kDa	0	11	0	11	0	2
Phosphoglycerate kinase, chloroplastic OS=Triticum aestivum OX=4565 PE=2 SV=1	P12782	50 kDa	2	8	1	9	1	4
Cluster of Polyadenylate-binding protein OS=Triticum aestivum OX=4565 PE=3 SV=1 (A0A3B6TF31)	A0A3B6TF31	69 kDa	2	0	4	4	4	1
Cluster of Glucose-6-phosphate isomerase OS=Triticum aestivum OX=4565 PE=3 SV=1 (A0A3B5XU98)	A0A3B5XU98 [2]	62 kDa	0	13	1	10	0	3
Cluster of Eukaryotic translation initiation factor 3 subunit B OS=Triticum aestivum OX=4565 PE=3 SV=1 (A0A3B6LV25)	A0A3B6LV25 [4]	83 kDa	0	3	5	6	1	4
Cluster of Peptidylprolyl isomerase OS=Triticum aestivum OX=4565 PE=4 SV=1 (A0A3B6RIA1)	A0A3B6RIA1 [3]	62 kDa	1	8	3	11	0	3
Cluster of 14-3-3 protein OS=Triticum aestivum OX=4565 PE=3 SV=1 (A0A3B6HZS8)	A0A3B6HZS8 [13]	28 kDa	1	8	1	11	0	6
Cluster of Peptidase_M24 domain-containing protein OS=Triticum aestivum OX=4565 PE=3 SV=1 (A0A3B5Y1K8)	A0A3B5Y1K8 [3]	43 kDa	0	3	2	10	2	3
KOW domain-containing protein OS=Triticum aestivum OX=4565 PE=3 SV=1	A0A3B6ENI7 (+2)	18 kDa	0	3	6	7	0	3
Uncharacterized protein OS=Triticum aestivum OX=4565 PE=3 SV=1	A0A3B6KBL0	22 kDa	1	4	3	7	0	6
Uncharacterized protein OS=Triticum aestivum OX=4565 PE=3 SV=1	A0A3B6LFF9	23 kDa	2	4	1	6	0	5
Cluster of CYTOSOL_AP domain-containing protein OS=Triticum aestivum OX=4565 PE=3 SV=1 (A0A3B6PSA8)	A0A3B6PSA8 [2]	55 kDa	1	10	1	9	0	3
Cluster of Tubulin beta chain OS=Triticum aestivum OX=4565 PE=3 SV=1 (A0A3B6KNM8)	A0A3B6KNM8 [8]	47 kDa	0	5	2	6	0	3
Cluster of EF-hand domain-containing protein OS=Triticum aestivum OX=4565 PE=4 SV=1 (A0A3B6SG35)	A0A3B6SG35 [11]	160 kDa	0	0	0	5	0	2
Cluster of Eukaryotic translation initiation factor 5B OS=Triticum aestivum OX=4565 PE=4 SV=1 (A0A3B5Z6Z8)	A0A3B5Z6Z8 [2]	143 kDa	0	0	0	10	0	7
Succinate--CoA ligase [ADP-forming] subunit beta, mitochondrial OS=Triticum aestivum OX=4565 PE=3 SV=1	A0A1D6B2M0 (+1)	45 kDa	1	11	1	9	0	4
Peptidase_M3 domain-containing protein OS=Triticum aestivum OX=4565 PE=3 SV=1	A0A3B6NXL4 (+2)	85 kDa	2	6	1	9	0	4
Cluster of Uncharacterized protein OS=Triticum aestivum OX=4565 PE=4 SV=1 (A0A3B6ILV9)	A0A3B6ILV9 [4]	69 kDa	1	2	2	3	0	4
Cluster of Aminopeptidase OS=Triticum aestivum OX=4565 PE=3 SV=1 (A0A3B6PKK3)	A0A3B6PKK3 [2]	98 kDa	1	12	0	9	0	2
Cluster of Uncharacterized protein OS=Triticum aestivum OX=4565 PE=3 SV=1 (A0A3B5ZSZ8)	A0A3B5ZSZ8 [6]	73 kDa	0	12	1	7	0	3
Cluster of Uncharacterized protein OS=Triticum aestivum OX=4565 PE=3 SV=1 (A0A3B5XVD9)	A0A3B5XVD9 [3]	34 kDa	2	9	0	10	0	3
Cluster of S-(hydroxymethyl)glutathione dehydrogenase OS=Triticum aestivum OX=4565 PE=3 SV=1 (A0A3B6PS43)	A0A3B6PS43 [3]	41 kDa	1	8	1	6	0	4
Histidine--tRNA ligase OS=Triticum aestivum OX=4565 PE=3 SV=1	A0A3B5ZRV5	95 kDa	1	6	0	5	1	3
Cluster of Eukaryotic translation initiation factor 3 subunit I OS=Triticum aestivum OX=4565 PE=3 SV=1 (A0A3B6HW61)	A0A3B6HW61 [6]	38 kDa	2	3	3	2	2	2
Cluster of Proteasome subunit beta OS=Triticum aestivum OX=4565 PE=3 SV=1 (A0A3B6I3L9)	A0A3B6I3L9 [2]	29 kDa	0	10	1	6	0	2
Cluster of Sucrose synthase OS=Triticum aestivum OX=4565 PE=3 SV=1 (A0A3B6REV7)	A0A3B6REV7 [5]	92 kDa	0	9	1	8	0	2
Ribosomal_L16 domain-containing protein OS=Triticum aestivum OX=4565 PE=3 SV=1	A0A0C4BIR6	25 kDa	0	3	4	6	0	1
Uncharacterized protein OS=Triticum aestivum OX=4565 PE=3 SV=1	A0A3B6HYB1 (+3)	61 kDa	0	8	0	5	0	6
26S proteasome non-ATPase regulatory subunit 2 homolog OS=Triticum aestivum OX=4565 PE=3 SV=1	A0A3B6NLY5 (+4)	97 kDa	0	3	1	5	0	2
Ribosomal_L2_C domain-containing protein OS=Triticum aestivum OX=4565 PE=3 SV=1	W5ECL2	28 kDa	0	2	1	7	0	2
Cluster of Proteasome subunit alpha type OS=Triticum aestivum OX=4565 PE=3 SV=1 (W5G0E6)	W5G0E6 [2]	27 kDa	2	5	4	5	0	4
Cluster of Dihydrolipoyl dehydrogenase OS=Triticum aestivum OX=4565 PE=3 SV=1 (A0A3B5XW54)	A0A3B5XW54 [2]	53 kDa	0	8	2	6	0	2
Cluster of Glutamate decarboxylase OS=Triticum aestivum OX=4565 PE=3 SV=1 (A0A3B6FIG0)	A0A3B6FIG0 [2]	54 kDa	0	10	0	7	0	5
Cluster of EF1_GNE domain-containing protein OS=Triticum aestivum OX=4565 PE=3 SV=1 (A0A3B6DB70)	A0A3B6DB70 [3]	23 kDa	0	8	2	9	0	1
Cluster of 40S ribosomal protein SA OS=Triticum aestivum OX=4565 PE=3 SV=1 (A0A3B6HQ62)	A0A3B6HQ62 [5]	32 kDa	0	7	3	9	0	3
Transaldolase OS=Triticum aestivum OX=4565 PE=3 SV=1	A0A3B6H0W1	44 kDa	2	6	2	6	0	4
DNA damage-binding protein 1 OS=Triticum aestivum OX=4565 PE=3 SV=1	A0A3B6U312	122 kDa	0	2	0	6	0	4
Cluster of AAI domain-containing protein OS=Triticum aestivum OX=4565 PE=3 SV=1 (A0A3B6GNC7)	A0A3B6GNC7 [5]	17 kDa	1	4	1	5	0	6
Cluster of Eukaryotic translation initiation factor 3 subunit C OS=Triticum aestivum OX=4565 PE=3 SV=1 (A0A3B6KJI3)	A0A3B6KJI3	105 kDa	0	0	2	8	0	2
Cluster of Single-stranded nucleic acid binding protein OS=Triticum aestivum OX=4565 GN=whGRP-1 PE=2 SV=1 (Q41518)	Q41518 [2]	16 kDa	0	6	1	6	0	1
Cluster of RRM domain-containing protein OS=Triticum aestivum OX=4565 PE=4 SV=1 (A0A3B6JDJ0)	A0A3B6JDJ0 [2]	16 kDa	0	4	1	6	0	6
Cluster of Aldedh domain-containing protein OS=Triticum aestivum OX=4565 PE=3 SV=1 (A0A3B6RGM5)	A0A3B6RGM5 [2]	59 kDa	1	6	0	8	0	2
Cluster of Putative alanine aminotransferase OS=Triticum aestivum OX=4565 GN=TaCQsd1 PE=2 SV=1 (W5FWW5)	W5FWW5 [2]	55 kDa	1	7	1	4	0	4
Cluster of Uncharacterized protein OS=Triticum aestivum OX=4565 PE=4 SV=1 (A0A3B6GTS5)	A0A3B6GTS5	12 kDa	0	4	2	6	0	5
Pyruvate kinase OS=Triticum aestivum OX=4565 PE=3 SV=1	A0A3B6LJK2	57 kDa	1	7	1	4	0	2
Uncharacterized protein OS=Triticum aestivum OX=4565 PE=3 SV=1	A0A3B6RNX6 (+1)	12 kDa	0	4	2	5	0	3
SHSP domain-containing protein OS=Triticum aestivum OX=4565 PE=3 SV=1	A0A1D5WXA1 (+3)	17 kDa	2	4	1	1	0	2
Cluster of Adenylyl cyclase-associated protein OS=Triticum aestivum OX=4565 PE=3 SV=1 (A0A3B6JFR8)	A0A3B6JFR8 [2]	51 kDa	0	1	1	5	1	2
Cluster of Uncharacterized protein OS=Triticum aestivum OX=4565 PE=3 SV=1 (A0A3B6C0D4)	A0A3B6C0D4 [3]	14 kDa	0	4	1	4	0	6
Cluster of Uncharacterized protein OS=Triticum aestivum OX=4565 PE=4 SV=1 (A0A3B6CE61)	A0A3B6CE61 [3]	34 kDa	2	4	1	6	0	7
Cluster of Guanosine nucleotide diphosphate dissociation inhibitor OS=Triticum aestivum OX=4565 PE=3 SV=1 (A0A3B5Y268)	A0A3B5Y268 [2]	50 kDa	0	7	2	4	0	4
Cluster of Alpha-amylase/trypsin inhibitor CM16 OS=Triticum aestivum OX=4565 PE=1 SV=1 (P16159)	P16159	16 kDa	1	3	0	5	0	3
Cluster of Uncharacterized protein OS=Triticum aestivum OX=4565 PE=4 SV=1 (A0A3B6ISM7)	A0A3B6ISM7 [4]	84 kDa	0	4	1	6	1	2
Cluster of Aspartate aminotransferase OS=Triticum aestivum OX=4565 GN=TRAES_3BF023000050CFD_c1 PE=3 SV=1 (A0A077S3V2)	A0A077S3V2 [2]	50 kDa	1	6	1	5	0	1

Cluster of Aldehyde dehydrogenase (NAD(+)) OS=Triticum aestivum OX=4565 PE=3 SV=1 (A0A3B6LKM3)	A0A3B6LKM3 [3]	54 kDa	0	7	0	3	0	4
Peroxiredoxin Q, chloroplastic OS=Triticum aestivum OX=4565 GN=PRX1 PE=2 SV=1	Q5S1S6	23 kDa	0	6	0	6	0	5
Cluster of Protein-synthesizing GTPase OS=Triticum aestivum OX=4565 PE=4 SV=1 (A0A3B5Y477)	A0A3B5Y477 [2]	51 kDa	0	0	1	7	0	4
Cluster of Aconitate hydratase OS=Triticum aestivum OX=4565 PE=3 SV=1 (A0A3B6PT84)	A0A3B6PT84 [4]	106 kDa	0	10	0	9	0	1
Cluster of Protein-serine/threonine phosphatase OS=Triticum aestivum OX=4565 PE=3 SV=1 (A0A3B6RL15)	A0A3B6RL15 [2]	34 kDa	0	8	0	4	0	3
Cluster of Uncharacterized protein OS=Triticum aestivum OX=4565 PE=3 SV=1 (A0A3B5Y2A7)	A0A3B5Y2A7 [3]	12 kDa	2	5	3	6	0	1
Cluster of Uncharacterized protein OS=Triticum aestivum OX=4565 PE=3 SV=1 (A0A3B6LUH0)	A0A3B6LUH0	145 kDa	1	6	0	6	0	1
Cluster of Protein-serine/threonine phosphatase OS=Triticum aestivum OX=4565 PE=4 SV=1 (A0A3B6AWD3)	A0A3B6AWD3 [2]	34 kDa	2	6	1	3	0	2
Cluster of Uncharacterized protein OS=Triticum aestivum OX=4565 PE=4 SV=1 (A0A3B6EHK7)	A0A3B6EHK7 [5]	36 kDa	0	6	0	5	0	1
Cluster of Aminotran_1_2 domain-containing protein OS=Triticum aestivum OX=4565 PE=4 SV=1 (A0A3B5XVF6)	A0A3B5XVF6 [5]	53 kDa	0	7	1	4	0	3
Cluster of SERPIN domain-containing protein OS=Triticum aestivum OX=4565 PE=3 SV=1 (A0A3B6JDS9)	A0A3B6JDS9 [6]	43 kDa	0	7	1	7	0	4
Cluster of Thioredox_DsbH domain-containing protein OS=Triticum aestivum OX=4565 PE=4 SV=1 (A0A3B6H4N7)	A0A3B6H4N7	90 kDa	0	5	0	6	0	4
6-phosphogluconate dehydrogenase, decarboxylating OS=Triticum aestivum OX=4565 PE=3 SV=1	A0A3B6H679 (+5)	54 kDa	0	6	0	6	0	3
Malate dehydrogenase OS=Triticum aestivum OX=4565 PE=3 SV=1	A0A3B5Y7Z1 (+2)	36 kDa	1	4	2	6	0	2
Cluster of Uncharacterized protein OS=Triticum aestivum OX=4565 PE=4 SV=1 (A0A3B6SGP9)	A0A3B6SGP9 [4]	46 kDa	0	7	0	8	0	1
Cluster of Glycosyltransferase OS=Triticum aestivum OX=4565 PE=3 SV=1 (A0A3B6DAQ7)	A0A3B6DAQ7 [2]	51 kDa	1	7	0	5	0	3
Cluster of Uncharacterized protein OS=Triticum aestivum OX=4565 PE=3 SV=1 (A0A3B6D8U9)	A0A3B6D8U9 [4]	62 kDa	0	0	1	2	0	0
60S acidic ribosomal protein P0 OS=Triticum aestivum OX=4565 PE=3 SV=1	A0A3B6RK63 (+2)	35 kDa	0	2	3	4	0	3
Cluster of Uncharacterized protein OS=Triticum aestivum OX=4565 GN=TRAES_3BF046400050CFD_c1 PE=4 SV=1 (A0A077S144)	A0A077S144 [3]	44 kDa	2	0	0	4	1	1
Cluster of HABP4_PAI-RBP1 domain-containing protein OS=Triticum aestivum OX=4565 PE=4 SV=1 (A0A3B5ZZA0)	A0A3B5ZZA0 [2]	40 kDa	2	2	1	1	0	3
Cluster of Rab protein OS=Triticum aestivum OX=4565 GN=rab 15B PE=2 SV=1 (Q41579)	Q41579 [2]	23 kDa	2	6	2	4	0	3
Cluster of 60S ribosomal protein L18a OS=Triticum aestivum OX=4565 PE=3 SV=1 (A0A1D5SRX8)	A0A1D5SRX8	21 kDa	0	3	0	3	0	2
Cluster of AAI domain-containing protein OS=Triticum aestivum OX=4565 PE=4 SV=1 (A0A3B6TIG9)	A0A3B6TIG9 [4]	19 kDa	0	5	1	4	0	2
Cluster of Eukaryotic translation initiation factor 3 subunit A OS=Triticum aestivum OX=4565 PE=3 SV=1 (A0A3B6GJX5)	A0A3B6GJX5 [3]	115 kDa	0	0	1	6	0	0
Cluster of WSI18 protein OS=Triticum aestivum OX=4565 PE=2 SV=1 (A0A0F7GA49)	A0A0F7GA49 [2]	23 kDa	0	8	1	3	0	4
Cluster of Uncharacterized protein OS=Triticum aestivum OX=4565 PE=3 SV=1 (A0A3B6ITL4)	A0A3B6ITL4 [2]	63 kDa	0	5	1	9	0	2
Cluster of Aconitate hydratase OS=Triticum aestivum OX=4565 PE=3 SV=1 (A0A3B6KT79)	A0A3B6KT79	107 kDa	0	7	0	5	0	2
40S ribosomal protein S8 OS=Triticum aestivum OX=4565 GN=CAMPLR22A2D_LOCUS2741 PE=3 SV=1	A0A1D5UKZ4	25 kDa	0	3	1	5	0	0
Uncharacterized protein OS=Triticum aestivum OX=4565 PE=4 SV=1	A0A3B6RKG6 (+2)	52 kDa	1	1	3	5	0	1
Cluster of Tubulin alpha chain OS=Triticum aestivum OX=4565 PE=3 SV=1 (A0A1D5SH28)	A0A1D5SH28 [7]	43 kDa	0	1	1	3	0	3
Cluster of Superoxide dismutase OS=Triticum aestivum OX=4565 GN=SOD3.1 PE=2 SV=1 (Q96185)	Q96185 [2]	25 kDa	1	7	0	3	0	1
Cluster of Uncharacterized protein OS=Triticum aestivum OX=4565 PE=3 SV=1 (A0A3B6PJV0)	A0A3B6PJV0 [3]	24 kDa	0	1	1	5	0	2
Acetyl-CoA carboxylase OS=Triticum aestivum OX=4565 GN=Acc-1 PE=4 SV=1	B2ZGL2	255 kDa	1	1	0	5	1	2
Uncharacterized protein OS=Triticum aestivum OX=4565 PE=4 SV=1	A0A3B6EIH6 (+6)	19 kDa	1	3	4	4	0	3
Superoxide dismutase [Cu-Zn] OS=Triticum aestivum OX=4565 PE=3 SV=1	A0A3B6ASA5 (+2)	16 kDa	1	2	2	2	1	2
Pyruvate decarboxylase OS=Triticum aestivum OX=4565 PE=3 SV=1	A0A3B6AS49 (+1)	64 kDa	1	4	1	4	0	2
VARLMGL domain-containing protein OS=Triticum aestivum OX=4565 PE=4 SV=1	A0A3B6MYJ6	129 kDa	0	6	1	5	0	5
Cluster of Uncharacterized protein OS=Triticum aestivum OX=4565 PE=4 SV=1 (A0A3B6DF66)	A0A3B6DF66 [2]	51 kDa	1	1	2	3	0	0
Cluster of 3-hydroxyacyl-CoA dehydrogenase OS=Triticum aestivum OX=4565 PE=3 SV=1 (A0A3B6PIU6)	A0A3B6PIU6 [2]	79 kDa	0	0	0	7	1	0
Uncharacterized protein OS=Triticum aestivum OX=4565 GN=TRAES_3BF060500310CFD_c1 PE=3 SV=1	A0A077RTE5 (+4)	21 kDa	0	4	0	6	0	0
V-ATPase 69 kDa subunit OS=Triticum aestivum OX=4565 PE=3 SV=1	A0A341Y4U2	68 kDa	0	4	1	4	0	2
Methylmalonate-semialdehyde dehydrogenase (CoA acylating) OS=Triticum aestivum OX=4565 PE=3 SV=1	A0A3B6B5W5 (+2)	57 kDa	0	5	0	5	0	1
Pyruvate kinase OS=Triticum aestivum OX=4565 PE=3 SV=1	A0A3B6CIR4	55 kDa	0	5	0	2	0	2
Phosphoglycerate mutase (2,3-diphosphoglycerate-independent) OS=Triticum aestivum OX=4565 PE=3 SV=1	A0A3B6EK73 (+1)	61 kDa	0	9	0	5	0	0
Isocitrate dehydrogenase [NADP] OS=Triticum aestivum OX=4565 PE=3 SV=1	A0A3B6FMD0 (+1)	51 kDa	1	4	1	4	0	1
sp|ALBU_BOVIN|	sp|ALBU_BOVIN|	69 kDa	0	3	0	3	0	4
Malate dehydrogenase OS=Triticum aestivum OX=4565 GN=TRAES_3BF087800010CFD_c1 PE=3 SV=1	A0A077RTG8 (+1)	35 kDa	0	6	1	4	0	0
Cluster of CCT-theta OS=Triticum aestivum OX=4565 PE=3 SV=1 (A0A3B6IQS5)	A0A3B6IQS5	58 kDa	0	3	1	4	0	2
Cluster of Succinate-semialdehyde dehydrogenase OS=Triticum aestivum OX=4565 PE=3 SV=1 (A0A3B6PJL2)	A0A3B6PJL2	56 kDa	1	9	0	3	0	0
Uncharacterized protein OS=Triticum aestivum OX=4565 PE=3 SV=1	A0A3B6NXW9 (+1)	59 kDa	0	4	0	4	0	1
CBS domain-containing protein OS=Triticum aestivum OX=4565 PE=4 SV=1	A0A3B6PJ20 (+1)	47 kDa	0	6	0	5	0	1
Ribosomal_L18e/L15P domain-containing protein OS=Triticum aestivum OX=4565 PE=3 SV=1	A0A341P0J7	21 kDa	0	0	0	6	0	3
Eukaryotic translation initiation factor 3 subunit G OS=Triticum aestivum OX=4565 PE=3 SV=1	A0A3B6NWB4 (+1)	31 kDa	0	2	2	2	0	2
Cluster of Uncharacterized protein OS=Triticum aestivum OX=4565 PE=4 SV=1 (A0A3B6JHB6)	A0A3B6JHB6	55 kDa	0	4	1	4	0	1
Cluster of Non-specific lipid-transfer protein OS=Triticum aestivum OX=4565 PE=3 SV=1 (A0A3B6HVU8)	A0A3B6HVU8 [2]	12 kDa	0	5	0	4	0	4
Ketol-acid reductoisomerase OS=Triticum aestivum OX=4565 PE=3 SV=1	A0A3B5Y7Y1 (+3)	63 kDa	0	6	0	4	0	0
OMPdecase OS=Triticum aestivum OX=4565 PE=3 SV=1	A0A3B6H1U9 (+1)	50 kDa	0	5	1	3	0	1
Proliferating cell nuclear antigen OS=Triticum aestivum OX=4565 PE=3 SV=1	A0A3B6PT03 (+2)	29 kDa	0	4	0	6	0	1
HATPase_c domain-containing protein OS=Triticum aestivum OX=4565 PE=3 SV=1	A0A3B6RS92 (+1)	93 kDa	0	4	0	7	0	0
Uncharacterized protein OS=Triticum aestivum OX=4565 PE=3 SV=1	A0A3B6MP76 (+2)	20 kDa	0	6	0	4	0	1
Transketolase OS=Triticum aestivum OX=4565 GN=CAMPLR22A2D_LOCUS615 PE=3 SV=1	A0A1D5UVU1	76 kDa	0	3	0	4	0	3
Chitinase OS=Triticum aestivum OX=4565 GN=CAMPLR22A2D_LOCUS3502 PE=4 SV=1	A0A1D5UH99 (+1)	29 kDa	0	2	0	4	0	2
Peroxidase OS=Triticum aestivum OX=4565 PE=3 SV=1	A0A3B6H2L0	39 kDa	0	3	0	7	0	2
Cluster of sp|RS27A_HUMAN|	sp|RS27A_HUMAN| [4]	18 kDa	1	3	1	2	2	0
Cluster of 60S ribosomal protein L13 OS=Triticum aestivum OX=4565 PE=3 SV=1 (A0A3B6RJP2)	A0A3B6RJP2	24 kDa	0	1	2	1	0	1
Cluster of Ribosomal_L28e domain-containing protein OS=Triticum aestivum OX=4565 PE=3 SV=1 (A0A3B5Z336)	A0A3B5Z336	17 kDa	0	1	3	2	0	1
Cluster of Eukaryotic translation initiation factor 3 subunit L OS=Triticum aestivum OX=4565 PE=3 SV=1 (A0A3B6EFG4)	A0A3B6EFG4 [3]	61 kDa	0	2	1	0	1	2
Cluster of M20_dimer domain-containing protein OS=Triticum aestivum OX=4565 PE=4 SV=1 (A0A3B6PNX4)	A0A3B6PNX4 [3]	49 kDa	1	4	2	4	0	2
Cluster of ATP-dependent 6-phosphofructokinase OS=Triticum aestivum OX=4565 GN=PFK PE=3 SV=1 (A0A1D5UP90)	A0A1D5UP90 [3]	51 kDa	0	3	1	5	0	3
Cluster of Proteasome subunit alpha type OS=Triticum aestivum OX=4565 PE=3 SV=1 (A0A3B6HSJ9)	A0A3B6HSJ9 [4]	26 kDa	0	6	0	7	0	2
Uncharacterized protein OS=Triticum aestivum OX=4565 PE=4 SV=1	A0A3B6LFC8	24 kDa	1	4	0	1	0	2
40S ribosomal protein S4 OS=Triticum aestivum OX=4565 PE=3 SV=1	A0A1D5SLI4	30 kDa	0	2	1	3	0	1
SOR_SNZ domain-containing protein OS=Triticum aestivum OX=4565 PE=3 SV=1	A0A3B6HWQ2 (+2)	33 kDa	1	5	0	4	0	1

Uncharacterized protein OS=Triticum aestivum OX=4565 PE=4 SV=1	A0A3B6GXD3	8 kDa	0	4	1	4	0	3
Genome assembly, chromosome: II OS=Triticum aestivum OX=4565 GN=CAMPLR22A2D_LOCUS3288 PE=3 SV=1	A0A1D5UIJ5 (+2)	55 kDa	0	4	1	3	0	3
AAI domain-containing protein OS=Triticum aestivum OX=4565 GN=TRAES_3BF068600010CFD_c1 PE=3 SV=1	W5D003	16 kDa	0	4	0	3	0	4
Uncharacterized protein OS=Triticum aestivum OX=4565 PE=3 SV=1	A0A3B6TD77	105 kDa	0	3	1	1	1	0
Cluster of Uncharacterized protein OS=Triticum aestivum OX=4565 PE=4 SV=1 (A0A3B6A134)	A0A3B6A134	58 kDa	0	4	0	1	0	3
Cluster of Epimerase domain-containing protein OS=Triticum aestivum OX=4565 PE=4 SV=1 (A0A3B6RK39)	A0A3B6RK39 [4]	35 kDa	1	6	1	3	0	1
Cluster of Genome assembly, chromosome: II OS=Triticum aestivum OX=4565 GN=CAMPLR22A2D_LOCUS2947 PE=3 SV=1 (A0A0C4BIW4)	A0A0C4BIW4	16 kDa	0	1	0	1	0	1
Cluster of Uncharacterized protein OS=Triticum aestivum OX=4565 PE=3 SV=1 (W5FEV1)	W5FEV1	28 kDa	0	0	2	4	0	1
Proteasome subunit alpha type OS=Triticum aestivum OX=4565 PE=3 SV=1	A0A3B6NM56 (+2)	31 kDa	0	4	0	5	0	0
Pyrophosphate--fructose 6-phosphate 1-phosphotransferase subunit alpha OS=Triticum aestivum OX=4565 GN=PFP-ALPHA PE=3 SV=1	A0A3B6NTY7 (+1)	63 kDa	0	6	0	4	0	1
CCT-eta OS=Triticum aestivum OX=4565 PE=3 SV=1	A0A3B6PKI6	60 kDa	0	4	0	7	0	1
Uncharacterized protein OS=Triticum aestivum OX=4565 PE=3 SV=1	A0A3B6NQX8 (+3)	18 kDa	0	3	2	2	0	2
Uncharacterized protein OS=Triticum aestivum OX=4565 PE=3 SV=1	W5FSM8	19 kDa	0	2	1	3	0	0
Uncharacterized protein OS=Triticum aestivum OX=4565 PE=3 SV=1	A0A3B6LFE7	23 kDa	0	0	1	3	0	2
Cluster of Citrate synthase OS=Triticum aestivum OX=4565 PE=3 SV=1 (A0A3B6MYE4)	A0A3B6MYE4 [2]	55 kDa	0	0	1	3	2	2
Cluster of C2H2-type domain-containing protein OS=Triticum aestivum OX=4565 PE=3 SV=1 (A0A3B6A2W3)	A0A3B6A2W3 [2]	34 kDa	0	0	0	4	1	2
Cluster of Pyruvate dehydrogenase E1 component subunit beta OS=Triticum aestivum OX=4565 PE=4 SV=1 (A0A3B6REZ9)	A0A3B6REZ9 [3]	40 kDa	0	5	1	5	0	0
Cluster of PKS_ER domain-containing protein OS=Triticum aestivum OX=4565 PE=4 SV=1 (A0A3B6KLS3)	A0A3B6KLS3	38 kDa	0	4	0	3	0	0
Eukaryotic translation initiation factor 3 subunit H OS=Triticum aestivum OX=4565 PE=3 SV=1	A0A3B6C6H6	38 kDa	0	1	0	4	0	3
Valyl-tRNA synthetase OS=Triticum aestivum OX=4565 PE=3 SV=1	A0A3B6GP74 (+1)	120 kDa	1	4	1	6	0	1
Uncharacterized protein OS=Triticum aestivum OX=4565 PE=4 SV=1	A0A3B6TDT0	60 kDa	0	0	0	6	0	3
Cold regulated protein OS=Triticum aestivum OX=4565 GN=Wcor18 PE=2 SV=1	Q8H0B8	18 kDa	1	2	1	5	0	1
Uncharacterized protein OS=Triticum aestivum OX=4565 PE=4 SV=1	A0A3B6MMF1	18 kDa	1	4	0	2	1	1
RanBD1 domain-containing protein OS=Triticum aestivum OX=4565 PE=4 SV=1	A0A3B5YXW1 (+1)	24 kDa	1	3	1	2	0	1
Serine hydroxymethyltransferase OS=Triticum aestivum OX=4565 GN=CAMPLR22A2D_LOCUS4912 PE=3 SV=1	A0A1D5UPW5 (+1)	51 kDa	0	3	0	4	0	2
Ribosomal protein L17 OS=Triticum aestivum OX=4565 PE=1 SV=1	Q5I7K4	15 kDa	0	4	0	4	0	1
Lactamase_B domain-containing protein OS=Triticum aestivum OX=4565 PE=3 SV=1	A0A3B6HR92 (+5)	29 kDa	0	3	0	4	0	3
Proteasome subunit alpha type OS=Triticum aestivum OX=4565 PE=3 SV=1	A0A1D6CXT4 (+1)	27 kDa	2	2	2	4	0	2
Cluster of ABA inducible protein OS=Triticum aestivum OX=4565 GN=Wrab18 PE=2 SV=1 (Q7XAP5)	Q7XAP5 [2]	18 kDa	0	3	1	2	0	7
Cluster of T-complex protein 1 subunit gamma OS=Triticum aestivum OX=4565 PE=3 SV=1 (A0A3B6RHJ0)	A0A3B6RHJ0	61 kDa	0	5	0	5	0	0
Cluster of Diadenosine tetraphosphate synthetase OS=Triticum aestivum OX=4565 PE=3 SV=1 (A0A3B6TJ13)	A0A3B6TJ13 [2]	82 kDa	0	3	0	6	0	0
Aldedh domain-containing protein OS=Triticum aestivum OX=4565 PE=3 SV=1	A0A3B5Y4Y0 (+2)	61 kDa	0	6	0	4	0	1
Phospholipase D OS=Triticum aestivum OX=4565 PE=3 SV=1	A0A3B6EEL3	92 kDa	0	4	0	5	0	1
40S ribosomal protein S12 OS=Triticum aestivum OX=4565 PE=3 SV=1	W5FEZ3	15 kDa	0	3	0	3	0	0
Uncharacterized protein OS=Triticum aestivum OX=4565 PE=3 SV=1	A0A3B5XX39 (+3)	21 kDa	0	1	1	1	0	3
Polyadenylate-binding protein OS=Triticum aestivum OX=4565 GN=CAMPLR22A2D_LOCUS3604 PE=3 SV=1	A0A2X0S855 (+2)	72 kDa	0	1	1	4	0	0
GTP-binding nuclear protein OS=Triticum aestivum OX=4565 GN=Ran1-3 PE=2 SV=1	Q944C6	25 kDa	0	3	1	3	0	0
Uncharacterized protein OS=Triticum aestivum OX=4565 PE=3 SV=1	A0A3B6SSH9	18 kDa	0	1	0	3	0	1
HABP4_PAI-RBP1 domain-containing protein OS=Triticum aestivum OX=4565 PE=4 SV=1	A0A3B6A3G3	40 kDa	1	1	1	3	0	3
Cluster of 40S ribosomal protein S6 OS=Triticum aestivum OX=4565 GN=CAMPLR22A2D_LOCUS542 PE=3 SV=1 (W5C8N6)	W5C8N6	28 kDa	0	1	1	0	0	1
Cluster of Importin subunit alpha OS=Triticum aestivum OX=4565 PE=3 SV=1 (A0A3B6FL79)	A0A3B6FL79 [3]	59 kDa	0	4	0	3	0	1
Cluster of Alanine--tRNA ligase OS=Triticum aestivum OX=4565 PE=3 SV=1 (A0A3B6HWM1)	A0A3B6HWM1 [3]	110 kDa	0	3	0	4	0	1
Cluster of AAA domain-containing protein OS=Triticum aestivum OX=4565 PE=4 SV=1 (A0A3B6GR56)	A0A3B6GR56 [6]	93 kDa	0	0	0	2	0	0
Cluster of Phosphoglycerate mutase (2,3-diphosphoglycerate-independent) OS=Triticum aestivum OX=4565 PE=3 SV=1 (A0A3B6IR14)	A0A3B6IR14	62 kDa	0	6	1	4	0	0
Ribosomal_L18e/L15P domain-containing protein OS=Triticum aestivum OX=4565 PE=3 SV=1	A0A3B6ATQ1 (+1)	21 kDa	0	0	0	2	0	1
Proteasome subunit alpha type OS=Triticum aestivum OX=4565 PE=3 SV=1	A0A3B6HR73 (+1)	27 kDa	1	3	0	4	0	0
SERPIN domain-containing protein OS=Triticum aestivum OX=4565 PE=3 SV=1	A0A3B6MWJ8 (+2)	43 kDa	0	4	1	3	0	1
FVE OS=Triticum aestivum OX=4565 GN=FVE PE=3 SV=1	A0A068B052 (+2)	50 kDa	0	5	0	2	0	1
ATP citrate synthase OS=Triticum aestivum OX=4565 PE=4 SV=1	A0A3B6EF27 (+5)	66 kDa	0	0	0	3	0	1
40S ribosomal protein S3a OS=Triticum aestivum OX=4565 PE=3 SV=1	A0A3B6IU74	30 kDa	0	0	2	4	0	0
Uncharacterized protein OS=Triticum aestivum OX=4565 PE=4 SV=1	A0A3B6KN68	25 kDa	0	4	0	3	0	0
Genome assembly, chromosome: II OS=Triticum aestivum OX=4565 GN=CAMPLR22A2D_LOCUS2763 PE=3 SV=1	W5C1E9	14 kDa	0	1	2	2	0	0
Genome assembly, chromosome: II OS=Triticum aestivum OX=4565 GN=CAMPLR22A2D_LOCUS3646 PE=3 SV=1	A0A2X0TY93	13 kDa	0	3	0	2	0	2
Dihydrolipoyl dehydrogenase OS=Triticum aestivum OX=4565 PE=3 SV=1	A0A3B5XVY7 (+4)	59 kDa	1	2	1	3	0	1
Ribosomal_L14e domain-containing protein OS=Triticum aestivum OX=4565 PE=3 SV=1	A0A096ULG3 (+2)	15 kDa	0	1	2	4	0	0
Alpha-amylase inhibitor 0.28 OS=Triticum aestivum OX=4565 GN=IMA1 PE=1 SV=3	P01083 (+1)	17 kDa	0	3	1	1	0	1
Serine hydroxymethyltransferase OS=Triticum aestivum OX=4565 PE=3 SV=1	A0A3B5YXP5 (+1)	58 kDa	0	2	1	3	0	1
Cluster of Tryptophanyl-tRNA synthetase OS=Triticum aestivum OX=4565 PE=3 SV=1 (A0A3B6KE85)	A0A3B6KE85 [4]	46 kDa	0	1	2	2	0	1
Cluster of 26S proteasome non-ATPase regulatory subunit 1 homolog OS=Triticum aestivum OX=4565 PE=3 SV=1 (A0A3B6CBX0)	A0A3B6CBX0 [2]	111 kDa	0	1	1	3	0	1
Cluster of Uncharacterized protein OS=Triticum aestivum OX=4565 PE=4 SV=1 (A0A3B6RKV2)	A0A3B6RKV2	11 kDa	1	2	2	1	0	1
Cluster of Formate dehydrogenase, mitochondrial OS=Triticum aestivum OX=4565 PE=3 SV=1 (A0A3B6RHA9)	A0A3B6RHA9 [2]	41 kDa	0	3	1	5	0	0
Cluster of Glutaredoxin-dependent peroxiredoxin OS=Triticum aestivum OX=4565 PE=3 SV=1 (A0A3B6SJF8)	A0A3B6SJF8	24 kDa	0	4	1	2	0	1
Cluster of Glutathione reductase OS=Triticum aestivum OX=4565 PE=3 SV=1 (A0A3B6PSL8)	A0A3B6PSL8	53 kDa	0	3	0	2	0	1
Cluster of AAA domain-containing protein OS=Triticum aestivum OX=4565 GN=TRAES_3BF045700010CFD_c1 PE=3 SV=1 (A0A077RXS4)	A0A077RXS4 [3]	48 kDa	0	1	0	2	0	1
Cluster of D-3-phosphoglycerate dehydrogenase OS=Triticum aestivum OX=4565 PE=3 SV=1 (A0A3B6B623)	A0A3B6B623	64 kDa	0	4	0	3	0	1
Cluster of CAF1C_H4-bd domain-containing protein OS=Triticum aestivum OX=4565 PE=3 SV=1 (A0A3B6HU10)	A0A3B6HU10	53 kDa	0	2	0	2	0	1
Cluster of Uncharacterized protein OS=Triticum aestivum OX=4565 PE=4 SV=1 (A0A3B6AV40)	A0A3B6AV40 [2]	48 kDa	0	2	1	2	0	1
Cluster of Dehydrin OS=Triticum aestivum OX=4565 GN=Wdhn1 PE=2 SV=1 (A0A0H4MAT1)	A0A0H4MAT1 [2]	14 kDa	0	4	1	3	0	3
Cluster of Tripeptidyl-peptidase II OS=Triticum aestivum OX=4565 PE=3 SV=1 (A0A3B6QFX2)	A0A3B6QFX2 [5]	140 kDa	0	5	0	4	0	2
Cluster of VWFA domain-containing protein OS=Triticum aestivum OX=4565 PE=3 SV=1 (A0A3B6JJG0)	A0A3B6JJG0 [2]	42 kDa	0	3	0	3	0	0
Cluster of 3-hydroxyacyl-CoA dehydrogenase OS=Triticum aestivum OX=4565 PE=3 SV=1 (A0A3B6EEU3)	A0A3B6EEU3 [3]	79 kDa	0	0	2	0	0	0
Asparagine--tRNA ligase OS=Triticum aestivum OX=4565 PE=3 SV=1	A0A3B5XWA7 (+1)	63 kDa	0	6	0	2	0	0
Carbamoyl-phosphate synthase (glutamine-hydrolyzing) OS=Triticum aestivum OX=4565 PE=3 SV=1	A0A3B6GW65 (+1)	128 kDa	0	2	0	5	0	1

Glucose-6-phosphate isomerase OS=Triticum aestivum OX=4565 PE=3 SV=1	A0A3B6KKG1 (+2)	67 kDa	0	5	0	4	0	0
Aldo_ket_red domain-containing protein OS=Triticum aestivum OX=4565 PE=3 SV=1	A0A3B6NXQ9 (+2)	42 kDa	0	0	0	0	3	0
S-formylglutathione hydrolase OS=Triticum aestivum OX=4565 PE=3 SV=1	A0A3B6EPQ6 (+3)	32 kDa	0	4	1	3	0	1
RRM domain-containing protein OS=Triticum aestivum OX=4565 PE=4 SV=1	A0A3B6H402	17 kDa	0	2	0	4	0	0
Sucrose synthase OS=Triticum aestivum OX=4565 PE=3 SV=1	A0A3B6JH89	92 kDa	0	4	0	3	0	0
Proteasome subunit beta OS=Triticum aestivum OX=4565 PE=3 SV=1	W5F826	24 kDa	0	3	0	3	0	2
NAC-A/B domain-containing protein OS=Triticum aestivum OX=4565 PE=4 SV=1	A0A3B5YY70 (+1)	22 kDa	0	2	1	3	0	0
Uncharacterized protein OS=Triticum aestivum OX=4565 PE=4 SV=1	A0A3B6B0J6 (+1)	34 kDa	0	2	1	1	0	2
Xylanase inhibitor protein 1 OS=Triticum aestivum OX=4565 GN=XIPI PE=1 SV=2	Q8L5C6	33 kDa	1	3	0	2	0	1
PITH domain-containing protein OS=Triticum aestivum OX=4565 GN=TRAES_3BF105700010CFD_c1 PE=4 SV=1	A0A077RSP3 (+2)	23 kDa	1	2	1	2	0	1
Ribosomal protein S20 OS=Triticum aestivum OX=4565 GN=rps20 PE=2 SV=1	U5HTD8	14 kDa	0	1	2	1	0	2
Uncharacterized protein OS=Triticum aestivum OX=4565 PE=4 SV=1	A0A3B5Y6R3	16 kDa	0	2	0	3	0	4
Cluster of Uncharacterized protein OS=Triticum aestivum OX=4565 PE=4 SV=1 (A0A341ZHG0)	A0A341ZHG0	24 kDa	1	1	0	1	0	2
Cluster of SHSP domain-containing protein OS=Triticum aestivum OX=4565 PE=3 SV=1 (A0A3B6HNY3)	A0A3B6HNY3	26 kDa	0	2	1	0	0	0
Cluster of Flap endonuclease 1 OS=Triticum aestivum OX=4565 GN=FEN1 PE=3 SV=1 (A0A3B5Y5H6)	A0A3B5Y5H6 [2]	43 kDa	1	1	2	2	0	1
Cluster of Uncharacterized protein OS=Triticum aestivum OX=4565 PE=4 SV=1 (A0A3B6LEY3)	A0A3B6LEY3	81 kDa	0	0	0	3	0	3
Cluster of Uncharacterized protein OS=Triticum aestivum OX=4565 PE=3 SV=1 (A0A3B6GQA1)	A0A3B6GQA1 [8]	96 kDa	0	3	0	1	0	0
Cluster of ATP-dependent 6-phosphofructokinase OS=Triticum aestivum OX=4565 GN=PFK PE=3 SV=1 (A0A3B5Z314)	A0A3B5Z314 [6]	62 kDa	0	2	0	2	0	0
Cluster of Methionine S-methyltransferase OS=Triticum aestivum OX=4565 PE=3 SV=1 (A0A3B5XTH3)	A0A3B5XTH3 [3]	120 kDa	0	3	0	4	0	1
Cluster of Transketolase OS=Triticum aestivum OX=4565 PE=3 SV=1 (A0A3B6TID0)	A0A3B6TID0 [3]	80 kDa	0	3	0	3	0	1
Cluster of PALP domain-containing protein OS=Triticum aestivum OX=4565 PE=3 SV=1 (A0A3B6FNB6)	A0A3B6FNB6 [2]	58 kDa	0	4	0	3	0	1
Cluster of Genome assembly, chromosome: II OS=Triticum aestivum OX=4565 GN=CAMPLR22A2D_LOCUS1640 PE=4 SV=1 (A0A2X0S376)	A0A2X0S376 [3]	49 kDa	0	2	0	5	0	0
Cluster of Aminopeptidase OS=Triticum aestivum OX=4565 PE=3 SV=1 (A0A3B6HXL9)	A0A3B6HXL9 [3]	99 kDa	0	6	0	5	0	0
Cluster of RRM domain-containing protein OS=Triticum aestivum OX=4565 GN=TRAES_3BF152900030CFD_c1 PE=4 SV=1 (W5CRM8)	W5CRM8 [2]	17 kDa	0	3	0	3	0	0
Uncharacterized protein OS=Triticum aestivum OX=4565 PE=3 SV=1	A0A3B6N086	55 kDa	0	2	0	4	0	0
Aminotran_1_2 domain-containing protein OS=Triticum aestivum OX=4565 PE=4 SV=1	A0A3B5YTN2	48 kDa	0	4	0	4	0	0
PABS domain-containing protein OS=Triticum aestivum OX=4565 PE=3 SV=1	A0A3B6RIK6	35 kDa	0	1	0	3	0	1
PCI domain-containing protein OS=Triticum aestivum OX=4565 PE=4 SV=1	A0A3B6NRL1	45 kDa	0	3	0	1	0	1
Glucose-1-phosphate adenylyltransferase large subunit, chloroplastic/amyloplastic (Fragment) OS=Triticum aestivum OX=4565 GN=AGA.7 PE=2 SV=1	P12300	56 kDa	0	2	1	2	0	2
Ribosomal protein L15 OS=Triticum aestivum OX=4565 PE=3 SV=1	A0A1D5UYA3 (+1)	22 kDa	0	1	1	2	0	0
Uncharacterized protein OS=Triticum aestivum OX=4565 PE=3 SV=1	A0A3B6HX49 (+1)	17 kDa	0	1	1	2	0	0
Coatomer subunit delta OS=Triticum aestivum OX=4565 PE=3 SV=1	A0A3B5Y1I8 (+2)	57 kDa	0	1	0	2	0	0
Uncharacterized protein OS=Triticum aestivum OX=4565 PE=4 SV=1	A0A3B6KDR2 (+2)	55 kDa	2	1	0	1	1	1
ADF-H domain-containing protein OS=Triticum aestivum OX=4565 PE=3 SV=1	A0A1D5ZLT8	16 kDa	0	1	0	3	0	1
Cluster of AAI domain-containing protein OS=Triticum aestivum OX=4565 PE=3 SV=1 (A0A3B6TFZ6)	A0A3B6TFZ6 [2]	16 kDa	0	2	0	1	0	0
Cluster of ADP/ATP translocase OS=Triticum aestivum OX=4565 PE=3 SV=1 (A0A3B6NT53)	A0A3B6NT53 [2]	41 kDa	0	3	0	0	0	1
Cluster of PfkB domain-containing protein OS=Triticum aestivum OX=4565 GN=TRAES_3BF078000040CFD_c1 PE=3 SV=1 (A0A077S6B3)	A0A077S6B3 [2]	35 kDa	0	2	1	1	0	1
Cluster of RNA helicase OS=Triticum aestivum OX=4565 PE=4 SV=1 (A0A3B6KP61)	A0A3B6KP61 [2]	67 kDa	0	0	1	1	0	2
Cluster of Leucyl-tRNA synthetase OS=Triticum aestivum OX=4565 PE=3 SV=1 (A0A3B6AUM2)	A0A3B6AUM2 [3]	123 kDa	0	2	1	3	0	0
Cluster of Genome assembly, chromosome: II OS=Triticum aestivum OX=4565 GN=CAMPLR22A2D_LOCUS1597 PE=3 SV=1 (W5BMW7)	W5BMW7 [3]	25 kDa	0	1	2	3	0	0
Cluster of Uncharacterized protein OS=Triticum aestivum OX=4565 PE=3 SV=1 (A0A3B6I180)	A0A3B6I180 [2]	41 kDa	1	2	0	3	0	1
Cluster of HABP4_PAI-RBP1 domain-containing protein OS=Triticum aestivum OX=4565 PE=4 SV=1 (A0A3B6EII0)	A0A3B6EII0 [2]	41 kDa	0	0	0	1	0	3
Cluster of S5 DRBM domain-containing protein OS=Triticum aestivum OX=4565 PE=3 SV=1 (A0A3B6KRQ3)	A0A3B6KRQ3 [3]	30 kDa	0	2	0	4	0	1
Cluster of Uncharacterized protein OS=Triticum aestivum OX=4565 PE=3 SV=1 (A0A3B6SFR4)	A0A3B6SFR4 [2]	39 kDa	1	3	0	3	0	1
Cluster of Cysteine synthase OS=Triticum aestivum OX=4565 PE=3 SV=1 (A0A3B6KBM0)	A0A3B6KBM0 [4]	45 kDa	0	4	1	2	0	0
Cluster of Formyltetrahydrofolate synthetase OS=Triticum aestivum OX=4565 PE=3 SV=1 (A0A3B6KJA2)	A0A3B6KJA2	76 kDa	1	2	0	2	0	0
Cluster of Translation machinery-associated protein 22 OS=Triticum aestivum OX=4565 GN=TRAES_3BF278300030CFD_c1 PE=3 SV=1 (A0A077RX59)	A0A077RX59 [4]	21 kDa	0	3	0	2	0	1
Cluster of Uncharacterized protein OS=Triticum aestivum OX=4565 PE=3 SV=1 (A0A3B6CG72)	A0A3B6CG72	47 kDa	0	2	0	3	0	1
Eukaryotic translation initiation factor 3 subunit D OS=Triticum aestivum OX=4565 PE=3 SV=1	A0A3B5Y6B4 (+2)	66 kDa	0	1	0	1	0	1
AAI domain-containing protein OS=Triticum aestivum OX=4565 PE=3 SV=1	A0A3B6JR20 (+1)	18 kDa	0	2	0	6	0	0
Uncharacterized protein OS=Triticum aestivum OX=4565 PE=4 SV=1	A0A3B6RIH4 (+7)	45 kDa	0	3	1	3	0	1
Uncharacterized protein OS=Triticum aestivum OX=4565 PE=3 SV=1	A0A3B6TPI1	17 kDa	0	0	0	1	0	3
Dihydrolipoyllysine-residue succinyltransferase OS=Triticum aestivum OX=4565 GN=CAMPLR22A2D_LOCUS339 PE=3 SV=1	A0A1D5UVY4 (+3)	49 kDa	0	0	0	2	0	1
Histidinol dehydrogenase, chloroplastic OS=Triticum aestivum OX=4565 PE=3 SV=1	A0A3B6B0C3 (+4)	51 kDa	0	2	2	1	0	1
Isoleucyl-tRNA synthetase OS=Triticum aestivum OX=4565 PE=3 SV=1	A0A3B6SU33	133 kDa	0	0	0	2	0	0
Uncharacterized protein OS=Triticum aestivum OX=4565 PE=3 SV=1	A0A3B6KN33 (+4)	32 kDa	0	2	1	2	0	1
Uncharacterized protein OS=Triticum aestivum OX=4565 PE=3 SV=1	A0A3B6LTN4 (+1)	24 kDa	0	0	0	3	0	0
Phenylalanyl-tRNA synthetase beta subunit OS=Triticum aestivum OX=4565 PE=3 SV=1	A0A3B5ZVL2 (+1)	67 kDa	0	2	0	1	0	0
Avenin-like b4 OS=Triticum aestivum OX=4565 PE=3 SV=1	A5A4L5 (+1)	33 kDa	1	3	0	1	0	0
Ribosomal protein L37 OS=Triticum aestivum OX=4565 PE=3 SV=1	A0A0C4BK42 (+1)	11 kDa	0	1	1	2	0	1
Cluster of HATPase_c domain-containing protein OS=Triticum aestivum OX=4565 PE=3 SV=1 (A0A3B6KBY5)	A0A3B6KBY5 [7]	91 kDa	0	3	1	3	0	0
Cluster of Uncharacterized protein OS=Triticum aestivum OX=4565 PE=3 SV=1 (A0A3B6FQD3)	A0A3B6FQD3	28 kDa	0	3	1	2	0	0
Cluster of Uncharacterized protein OS=Triticum aestivum OX=4565 PE=3 SV=1 (A0A3B6NV46)	A0A3B6NV46 [3]	48 kDa	0	4	0	3	0	0
Uncharacterized protein OS=Triticum aestivum OX=4565 PE=3 SV=1	A0A3B6KFG9	41 kDa	0	4	0	1	0	1
Uncharacterized protein OS=Triticum aestivum OX=4565 PE=4 SV=1	A0A3B6A005	28 kDa	0	3	1	2	0	0
Pyruvate dehydrogenase E1 component subunit alpha OS=Triticum aestivum OX=4565 PE=4 SV=1	A0A3B6TNY0	43 kDa	0	3	0	2	0	0
Glutaredoxin-dependent peroxiredoxin OS=Triticum aestivum OX=4565 PE=3 SV=1	A0A3B6ELY8 (+2)	17 kDa	1	2	1	2	0	0
Uncharacterized protein OS=Triticum aestivum OX=4565 PE=3 SV=1	A0A3B6LM62 (+2)	26 kDa	0	2	1	2	0	0
Proteasome subunit beta OS=Triticum aestivum OX=4565 PE=3 SV=1	A0A3B6JH57	27 kDa	0	2	0	2	0	0
Sucrose-phosphate phosphatase OS=Triticum aestivum OX=4565 PE=3 SV=1	A0A3B6LEP7	57 kDa	0	3	0	3	0	0
Eukaryotic translation initiation factor 3 subunit F OS=Triticum aestivum OX=4565 PE=3 SV=1	A0A3B6NM42 (+1)	32 kDa	0	0	0	2	0	1
Oleosin OS=Triticum aestivum OX=4565 GN=CAMPLR22A2D_LOCUS4168 PE=3 SV=1	A0A2X0SLS2 (+2)	16 kDa	1	1	1	1	1	1
Uncharacterized protein OS=Triticum aestivum OX=4565 PE=4 SV=1	A0A3B6CD73 (+2)	62 kDa	0	2	0	2	0	2

Proteasome subunit beta OS=Triticum aestivum OX=4565 PE=3 SV=1	A0A3B6I116 (+2)	23 kDa	0	3	0	2	0	1
Uncharacterized protein OS=Triticum aestivum OX=4565 PE=4 SV=1	A0A3B6LUL8	25 kDa	0	2	0	2	0	0
Uncharacterized protein OS=Triticum aestivum OX=4565 PE=3 SV=1	W5FVL1	16 kDa	0	1	1	2	0	0
Uncharacterized protein OS=Triticum aestivum OX=4565 PE=4 SV=1	A0A3B6NPL4 (+2)	70 kDa	1	1	0	2	0	1
Uncharacterized protein OS=Triticum aestivum OX=4565 PE=3 SV=1	A0A3B6REV0 (+1)	20 kDa	0	1	0	2	0	0
Uncharacterized protein OS=Triticum aestivum OX=4565 PE=4 SV=1	A0A3B6HS12	38 kDa	0	2	0	2	0	1
Protein phosphatase 2A structural subunit OS=Triticum aestivum OX=4565 PE=4 SV=1	A0A3B6KFF3 (+2)	64 kDa	0	1	0	0	0	0
Pyr_redox_2 domain-containing protein OS=Triticum aestivum OX=4565 PE=4 SV=1	A0A3B6RFJ3 (+2)	47 kDa	0	1	1	2	0	1
Cluster of PKS_ER domain-containing protein OS=Triticum aestivum OX=4565 PE=4 SV=1 (A0A3B6PT07)	A0A3B6PT07 [2]	34 kDa	1	2	1	1	0	2
Cluster of UDP-glucose 6-dehydrogenase OS=Triticum aestivum OX=4565 PE=3 SV=1 (A0A3B6HXF2)	A0A3B6HXF2 [3]	53 kDa	0	1	0	2	0	1
Cluster of E1 ubiquitin-activating enzyme OS=Triticum aestivum OX=4565 PE=3 SV=1 (A0A3B6KDT7)	A0A3B6KDT7 [9]	117 kDa	0	2	0	2	0	0
Cluster of Glutathione transferase OS=Triticum aestivum OX=4565 PE=3 SV=1 (A0A3B5XV82)	A0A3B5XV82 [3]	25 kDa	0	3	0	2	0	0
Cluster of Proteasome subunit alpha type OS=Triticum aestivum OX=4565 PE=3 SV=1 (A0A1D5YY58)	A0A1D5YY58	27 kDa	0	2	0	3	0	0
Cluster of Ribosomal protein L19 OS=Triticum aestivum OX=4565 PE=3 SV=1 (A0A1D5TPB6)	A0A1D5TPB6 [2]	24 kDa	0	0	0	2	0	0
Cluster of Arginyl-tRNA synthetase OS=Triticum aestivum OX=4565 PE=3 SV=1 (A0A3B5YU94)	A0A3B5YU94 [5]	68 kDa	0	4	0	2	0	0
Cluster of Glutamyl-tRNA synthetase OS=Triticum aestivum OX=4565 PE=3 SV=1 (A0A3B6A0F5)	A0A3B6A0F5 [2]	80 kDa	0	0	0	1	0	0
Cluster of Eukaryotic translation initiation factor 3 subunit M OS=Triticum aestivum OX=4565 PE=3 SV=1 (A0A3B6AQL4)	A0A3B6AQL4 [7]	46 kDa	0	0	0	2	0	0
Asparagine synthetase [glutamine-hydrolyzing] OS=Triticum aestivum OX=4565 GN=ASN2 PE=2 SV=1	A0A1D5VHV8 (+1)	65 kDa	0	3	0	2	0	0
Importin N-terminal domain-containing protein OS=Triticum aestivum OX=4565 PE=4 SV=1	A0A3B6KDT5 (+2)	96 kDa	0	2	0	4	0	1
Uncharacterized protein OS=Triticum aestivum OX=4565 PE=3 SV=1	A0A3B6RKF9 (+2)	79 kDa	0	3	0	2	0	0
Uncharacterized protein OS=Triticum aestivum OX=4565 PE=3 SV=1	A0A3B6U5E5	13 kDa	0	0	1	1	0	0
Aldo_ket_red domain-containing protein OS=Triticum aestivum OX=4565 PE=4 SV=1	A0A3B6QAB5	35 kDa	0	3	0	2	0	0
Uncharacterized protein OS=Triticum aestivum OX=4565 PE=4 SV=1	A0A3B6JKG3	34 kDa	0	2	0	1	0	1
Uncharacterized protein OS=Triticum aestivum OX=4565 PE=3 SV=1	A0A0C4BKI1	13 kDa	0	1	0	2	0	1
Ribosomal_S7 domain-containing protein OS=Triticum aestivum OX=4565 PE=3 SV=1	A0A3B6EC38 (+2)	22 kDa	0	2	0	2	0	1
Ribosomal_L18_c domain-containing protein OS=Triticum aestivum OX=4565 PE=3 SV=1	A0A3B6LRC5 (+1)	35 kDa	0	1	1	1	0	0
HECT-type E3 ubiquitin transferase OS=Triticum aestivum OX=4565 PE=4 SV=1	A0A3B6KDR3 (+1)	402 kDa	0	0	0	1	0	0
Glutaredoxin domain-containing protein OS=Triticum aestivum OX=4565 PE=3 SV=1	A0A3B6B2V5	13 kDa	1	1	0	2	0	1
ATP citrate synthase OS=Triticum aestivum OX=4565 PE=3 SV=1	A0A3B6KDV3 (+3)	47 kDa	0	2	0	2	0	1
Uncharacterized protein OS=Triticum aestivum OX=4565 PE=4 SV=1	A0A3B6INL0	23 kDa	0	2	0	0	0	0
40S ribosomal protein S25 OS=Triticum aestivum OX=4565 PE=3 SV=1	A0A3B6HY18 (+2)	11 kDa	0	1	1	2	0	2
Uncharacterized protein OS=Triticum aestivum OX=4565 PE=3 SV=1	A0A341T1J6 (+3)	11 kDa	0	1	2	2	0	0
Profilin OS=Triticum aestivum OX=4565 PE=3 SV=1	A0A3B6R960	14 kDa	0	2	0	2	0	0
AAA domain-containing protein OS=Triticum aestivum OX=4565 PE=3 SV=1	A0A0C4BK41 (+1)	48 kDa	0	0	0	2	0	0
Glutamate dehydrogenase OS=Triticum aestivum OX=4565 PE=3 SV=1	A0A3B6LUP3 (+3)	49 kDa	0	2	1	1	0	0
26S proteasome regulatory subunit RPN11 OS=Triticum aestivum OX=4565 PE=4 SV=1	A0A3B5Y160 (+3)	35 kDa	0	1	0	2	0	1
Genome assembly, chromosome: II OS=Triticum aestivum OX=4565 GN=CAMPLR22A2D_LOCUS2650 PE=3 SV=1	A0A2X0S5H9 (+2)	33 kDa	0	3	0	3	0	0
Cluster of rRNA N-glycosidase OS=Triticum aestivum OX=4565 PE=3 SV=1 (A0A3B6LWD5)	A0A3B6LWD5	31 kDa	1	0	1	2	0	0
Cluster of Uncharacterized protein OS=Triticum aestivum OX=4565 PE=4 SV=1 (A0A3B6IXS8)	A0A3B6IXS8	50 kDa	0	4	0	3	0	0
Cluster of Uncharacterized protein OS=Triticum aestivum OX=4565 PE=4 SV=1 (A0A3B6PQI2)	A0A3B6PQI2 [2]	46 kDa	0	0	0	1	0	0
Ribosomal_L23eN domain-containing protein OS=Triticum aestivum OX=4565 PE=3 SV=1	A0A3B6B204	20 kDa	0	0	1	1	0	0
Succinate--CoA ligase [ADP-forming] subunit alpha, mitochondrial OS=Triticum aestivum OX=4565 PE=3 SV=1	A0A3B6AVX5 (+2)	34 kDa	0	2	0	0	0	1
HP domain-containing protein OS=Triticum aestivum OX=4565 PE=4 SV=1	A0A3B6H1T3 (+1)	112 kDa	0	1	0	3	0	0
Fn3_like domain-containing protein OS=Triticum aestivum OX=4565 PE=3 SV=1	A0A3B6B6I7 (+3)	82 kDa	0	2	0	0	0	0
TRASH domain-containing protein OS=Triticum aestivum OX=4565 PE=3 SV=1	W5CDW4	18 kDa	0	1	0	2	0	1
Nudix hydrolase domain-containing protein OS=Triticum aestivum OX=4565 PE=4 SV=1	A0A3B6NVL3 (+5)	87 kDa	0	2	0	3	0	0
Vacuolar proton pump subunit B OS=Triticum aestivum OX=4565 PE=3 SV=1	A0A341SHX2 (+6)	54 kDa	0	2	0	2	0	1
Fructose-1,6-bisphosphate aldolase 19 OS=Triticum aestivum OX=4565 GN=FBA19 PE=3 SV=1	A0A1D6C057	148 kDa	0	2	0	2	0	1
Pyrophosphate--fructose 6-phosphate 1-phosphotransferase subunit beta OS=Triticum aestivum OX=4565 GN=PFP-BETA PE=3 SV=1	A0A1D6D1Q3 (+1)	61 kDa	1	1	0	2	0	1
40S ribosomal protein S26 OS=Triticum aestivum OX=4565 PE=3 SV=1	A0A3B6KQB8	15 kDa	0	1	1	1	0	0
Thioredoxin-dependent peroxiredoxin OS=Triticum aestivum OX=4565 GN=CAMPLR22A2D_LOCUS2968 PE=4 SV=1	A0A0C4BJ55 (+1)	28 kDa	0	1	1	3	0	0
Importin N-terminal domain-containing protein OS=Triticum aestivum OX=4565 PE=4 SV=1	A0A341P3K0 (+2)	96 kDa	0	1	0	2	0	0
Protein disulfide-isomerase OS=Triticum aestivum OX=4565 GN=pdil4-1 PE=2 SV=1	D8L9B3	40 kDa	0	3	0	1	0	0
Fumarylacetoacetase OS=Triticum aestivum OX=4565 PE=3 SV=1	A0A3B6NP66 (+1)	47 kDa	0	1	0	2	0	0
Uncharacterized protein OS=Triticum aestivum OX=4565 PE=3 SV=1	A0A3B6NM31 (+6)	45 kDa	0	2	1	1	0	1
Uncharacterized protein OS=Triticum aestivum OX=4565 PE=4 SV=1	A0A3B6HND4	56 kDa	0	2	0	1	0	0
Dihydrothymine dehydrogenase OS=Triticum aestivum OX=4565 PE=3 SV=1	A0A3B6NU95 (+1)	45 kDa	0	1	1	2	0	0
Cysteine proteinase inhibitor OS=Triticum aestivum OX=4565 PE=3 SV=1	A0A3B6GQ71 (+1)	27 kDa	0	2	0	2	0	1
Uncharacterized protein OS=Triticum aestivum OX=4565 PE=3 SV=1	A0A3B5XTY5 (+2)	38 kDa	0	1	0	2	0	1
30S ribosomal protein S4, chloroplastic OS=Triticum aestivum OX=4565 PE=3 SV=1	W5FPA7	22 kDa	0	1	0	1	0	0
Fructose-bisphosphate aldolase OS=Triticum aestivum OX=4565 PE=3 SV=1	A0A3B6E9X3	46 kDa	0	2	0	1	0	0
RNA-binding Ras-GAP SH3 binding protein OS=Triticum aestivum OX=4565 PE=4 SV=1	A0A3B6SKF0 (+3)	45 kDa	0	0	0	0	0	1
NAD(P)-bd_dom domain-containing protein OS=Triticum aestivum OX=4565 PE=4 SV=1	A0A3B6LWG8 (+1)	31 kDa	0	2	0	3	0	0
Malate dehydrogenase OS=Triticum aestivum OX=4565 PE=3 SV=1	A0A3B6SJ79	41 kDa	0	2	0	2	0	0
Uncharacterized protein OS=Triticum aestivum OX=4565 PE=3 SV=1	A0A3B6SI59	41 kDa	0	1	0	2	0	1
Chitinase OS=Triticum aestivum OX=4565 PE=4 SV=1	A0A3B6GXR9	33 kDa	0	2	0	2	0	0
Catalase OS=Triticum aestivum OX=4565 PE=3 SV=1	A0A3B6NJS8 (+4)	57 kDa	0	1	0	2	0	0
Glutamate dehydrogenase OS=Triticum aestivum OX=4565 PE=3 SV=1	A0A3B6B1Y1 (+2)	45 kDa	0	2	0	2	0	1
Alpha-1,4 glucan phosphorylase OS=Triticum aestivum OX=4565 PE=3 SV=1	A0A3B6EQH2	94 kDa	0	2	0	1	0	1
Glutathione peroxidase OS=Triticum aestivum OX=4565 GN=PHGPX6 PE=2 SV=1	A0A1D5UGB6	18 kDa	0	1	0	2	0	0
Cluster of AAI domain-containing protein OS=Triticum aestivum OX=4565 PE=3 SV=1 (A0A3B6KUN7)	A0A3B6KUN7 [2]	10 kDa	0	1	0	0	0	2
Cluster of Nop domain-containing protein OS=Triticum aestivum OX=4565 PE=3 SV=1 (A0A3B6C120)	A0A3B6C120	60 kDa	0	0	0	0	0	1
Cluster of Aha1_N domain-containing protein OS=Triticum aestivum OX=4565 PE=3 SV=1 (A0A3B6RJB7)	A0A3B6RJB7	39 kDa	0	3	0	1	0	1

Cluster of eIF2B_5 domain-containing protein OS=Triticum aestivum OX=4565 PE=3 SV=1 (A0A3B6IPF3)	A0A3B6IPF3 [2]	30 kDa	0	0	0	1	0	0
CN hydrolase domain-containing protein OS=Triticum aestivum OX=4565 PE=4 SV=1	A0A3B6KAS9 (+1)	33 kDa	0	1	0	3	0	0
Glucose-1-phosphate adenylyltransferase OS=Triticum aestivum OX=4565 PE=3 SV=1	A0A3B6RFZ5	56 kDa	0	3	0	1	0	0
Citrulline--aspartate ligase OS=Triticum aestivum OX=4565 PE=3 SV=1	A0A3B5Y2C2 (+3)	52 kDa	0	2	0	3	0	0
Uncharacterized protein OS=Triticum aestivum OX=4565 PE=4 SV=1	A0A3B6RJA4 (+2)	37 kDa	0	2	0	1	0	0
Succinate dehydrogenase [ubiquinone] flavoprotein subunit, mitochondrial OS=Triticum aestivum OX=4565 PE=3 SV=1	A0A3B6KQV6 (+2)	68 kDa	0	2	0	1	0	0
Eukaryotic translation initiation factor 3 subunit E OS=Triticum aestivum OX=4565 PE=3 SV=1	A0A3B6KK98 (+1)	51 kDa	0	0	1	1	0	1
Serine/threonine-protein phosphatase 5 OS=Triticum aestivum OX=4565 PE=3 SV=1	A0A3B6ED37 (+2)	54 kDa	0	2	1	1	0	0
Inositol-1,3,4-trisphosphate 5/6-kinase OS=Triticum aestivum OX=4565 PE=3 SV=1	A0A3B6LQC6 (+2)	55 kDa	0	2	0	1	0	1
ATP-dependent (S)-NAD(P)H-hydrate dehydratase OS=Triticum aestivum OX=4565 PE=3 SV=1	A0A3B6IMK6 (+2)	41 kDa	0	2	0	0	0	1
UDP-arabinopyranose mutase OS=Triticum aestivum OX=4565 PE=3 SV=1	A0A3B6B5P1 (+2)	39 kDa	0	2	0	1	0	0
Methanethiol oxidase OS=Triticum aestivum OX=4565 PE=3 SV=1	A0A3B6EN11	54 kDa	0	1	0	2	0	0
Sucrose-phosphate synthase OS=Triticum aestivum OX=4565 GN=TRAES_3BF045200020CFD_c1 PE=3 SV=1	A0A077RP15 (+3)	119 kDa	0	0	0	2	0	1
Epimerase domain-containing protein OS=Triticum aestivum OX=4565 PE=4 SV=1	A0A3B6LYJ8 (+1)	36 kDa	0	1	0	2	0	1
Abhydrolase_2 domain-containing protein OS=Triticum aestivum OX=4565 PE=4 SV=1	A0A3B6ECP2 (+4)	30 kDa	0	2	0	1	0	1
Ubiquitin receptor RAD23 OS=Triticum aestivum OX=4565 PE=3 SV=1	A0A3B6RGD2 (+1)	42 kDa	0	2	1	0	0	0
Genome assembly, chromosome: II OS=Triticum aestivum OX=4565 GN=CAMPLR22A2D_LOCUS742 PE=3 SV=1	A0A1D5U7G3 (+1)	50 kDa	0	0	0	1	0	0
Uncharacterized protein OS=Triticum aestivum OX=4565 PE=4 SV=1	A0A3B6ISY7 (+1)	68 kDa	0	2	0	0	0	0
Cluster of Uncharacterized protein OS=Triticum aestivum OX=4565 PE=3 SV=1 (A0A3B6JGH7)	A0A3B6JGH7	41 kDa	0	1	0	2	0	0
Cluster of 3-oxoacyl-[acyl-carrier-protein] synthase OS=Triticum aestivum OX=4565 PE=3 SV=1 (A0A3B6RBC5)	A0A3B6RBC5 [2]	48 kDa	0	0	0	1	0	1
Cluster of KH type-2 domain-containing protein OS=Triticum aestivum OX=4565 PE=3 SV=1 (W5I1R7)	W5I1R7	25 kDa	0	1	0	1	0	0
Cluster of Acetyl-CoA carboxylase OS=Triticum aestivum OX=4565 PE=4 SV=1 (A0A3B6FWS3)	A0A3B6FWS3 [3]	252 kDa	0	0	0	3	0	0
Cluster of Glutamate-1-semialdehyde 2,1-aminomutase OS=Triticum aestivum OX=4565 PE=3 SV=1 (A0A3B6TIX4)	A0A3B6TIX4 [2]	48 kDa	0	2	0	2	0	0
Cluster of Epimerase domain-containing protein OS=Triticum aestivum OX=4565 PE=4 SV=1 (A0A3B6KMD4)	A0A3B6KMD4	42 kDa	0	2	1	1	0	0
Cluster of Uncharacterized protein OS=Triticum aestivum OX=4565 PE=3 SV=1 (A0A3B5YWZ3)	A0A3B5YWZ3 [2]	16 kDa	0	0	1	0	0	0
Cluster of Threonyl-tRNA synthetase OS=Triticum aestivum OX=4565 GN=TRAES_3BF175400030CFD_c1 PE=3 SV=1 (A0A077RPZ7)	A0A077RPZ7 [2]	81 kDa	0	3	0	0	0	0
Glycosyltransferase OS=Triticum aestivum OX=4565 GN=TaGTd PE=2 SV=1	D5MTE2	50 kDa	0	1	0	2	0	0
Obg-like ATPase 1 OS=Triticum aestivum OX=4565 GN=TRAES_3BF155200010CFD_c1 PE=3 SV=1	A0A077RXR6 (+3)	44 kDa	0	0	0	3	0	0
Leuk-A4-hydro_C domain-containing protein OS=Triticum aestivum OX=4565 PE=3 SV=1	A0A3B6KSW7 (+2)	68 kDa	0	2	0	1	0	0
CULLIN_2 domain-containing protein OS=Triticum aestivum OX=4565 PE=3 SV=1	A0A3B6LUD9 (+2)	87 kDa	0	0	0	1	0	0
Uncharacterized protein OS=Triticum aestivum OX=4565 PE=3 SV=1	A0A3B5XXR5 (+1)	19 kDa	0	0	0	1	0	0
Uncharacterized protein OS=Triticum aestivum OX=4565 PE=4 SV=1	A0A3B6NNA4	30 kDa	0	1	0	2	0	1
PKS_ER domain-containing protein OS=Triticum aestivum OX=4565 PE=3 SV=1	A0A3B6TEG7	39 kDa	0	2	0	1	0	0
Uncharacterized protein OS=Triticum aestivum OX=4565 PE=4 SV=1	A0A3B6AS22 (+1)	59 kDa	0	2	0	1	0	1
A0A3B5Y8U0-DECOY	A0A3B5Y8U0-DECOY	?	0	2	0	2	0	0
CCT-epsilon OS=Triticum aestivum OX=4565 PE=3 SV=1	A0A3B6SG39 (+4)	57 kDa	0	1	0	1	0	0
RRM domain-containing protein OS=Triticum aestivum OX=4565 PE=4 SV=1	A0A3B6NSK1 (+5)	24 kDa	0	1	0	0	0	0
Peptidase_S9 domain-containing protein OS=Triticum aestivum OX=4565 PE=4 SV=1	A0A3B6RBU5 (+2)	79 kDa	0	1	0	2	0	0
Uncharacterized protein OS=Triticum aestivum OX=4565 PE=3 SV=1	A0A3B6RFN1	63 kDa	0	0	0	2	0	1
RRM domain-containing protein OS=Triticum aestivum OX=4565 PE=4 SV=1	A0A3B6I2W8 (+4)	28 kDa	0	2	0	0	0	1
30S ribosomal protein S8, chloroplastic OS=Triticum aestivum OX=4565 GN=TRAES_3BF117500090CFD_c1 PE=2 SV=1	E2F3W4	15 kDa	0	0	0	1	0	0
Proteasome subunit alpha type OS=Triticum aestivum OX=4565 PE=3 SV=1	A0A3B6FPZ0 (+4)	27 kDa	0	2	0	2	0	0
Cluster of Uncharacterized protein OS=Triticum aestivum OX=4565 PE=4 SV=1 (A0A3B6NSB1)	A0A3B6NSB1 [2]	173 kDa	0	0	0	2	0	0
Cluster of Usp domain-containing protein OS=Triticum aestivum OX=4565 PE=4 SV=1 (A0A3B5Z2J5)	A0A3B5Z2J5	28 kDa	0	2	0	0	0	0
Cluster of AIR synthase OS=Triticum aestivum OX=4565 PE=3 SV=1 (A0A3B6I1J0)	A0A3B6I1J0 [2]	40 kDa	0	3	0	1	0	1
Cluster of Uncharacterized protein OS=Triticum aestivum OX=4565 PE=4 SV=1 (A0A3B6KPZ7)	A0A3B6KPZ7 [3]	42 kDa	0	0	0	1	0	0
Cluster of HIT domain-containing protein OS=Triticum aestivum OX=4565 PE=4 SV=1 (W5E2R7)	W5E2R7	14 kDa	0	2	0	1	0	0
Cluster of Uncharacterized protein OS=Triticum aestivum OX=4565 PE=3 SV=1 (A0A3B5YZG5)	A0A3B5YZG5 [2]	115 kDa	0	2	0	0	0	0
Cluster of Uncharacterized protein OS=Triticum aestivum OX=4565 PE=4 SV=1 (A0A3B6A3D9)	A0A3B6A3D9	76 kDa	0	0	1	0	0	0
Cluster of WHy domain-containing protein OS=Triticum aestivum OX=4565 PE=3 SV=1 (A0A3B6GU21)	A0A3B6GU21 [2]	16 kDa	0	3	0	0	0	0
Cluster of Peroxidase OS=Triticum aestivum OX=4565 GN=TRAES_3BF171700070CFD_c1 PE=3 SV=1 (A0A077RPF9)	A0A077RPF9	36 kDa	0	0	0	1	0	0
Cluster of MBD6-5DS OS=Triticum aestivum OX=4565 PE=2 SV=1 (W5G255)	W5G255	44 kDa	0	1	0	0	0	2
Peptidase_S9 domain-containing protein OS=Triticum aestivum OX=4565 PE=4 SV=1	A0A3B6R9L3	39 kDa	0	1	0	2	0	0
Uncharacterized protein OS=Triticum aestivum OX=4565 PE=3 SV=1	A0A3B6KH02 (+3)	109 kDa	0	2	0	1	0	0
Genome assembly, chromosome: II OS=Triticum aestivum OX=4565 GN=CAMPLR22A2D_LOCUS410 PE=3 SV=1	A0A1D5UV66	61 kDa	0	2	0	1	0	0
A0A3B6NJT9-DECOY	A0A3B6NJT9-DECOY (+1)	?	0	1	0	2	0	0
Uncharacterized protein OS=Triticum aestivum OX=4565 PE=3 SV=1	A0A3B6HQN3 (+1)	57 kDa	0	2	0	1	0	0
Cluster of Hcy-binding domain-containing protein OS=Triticum aestivum OX=4565 PE=4 SV=1 (A0A3B6FU76)	A0A3B6FU76 [2]	38 kDa	0	0	0	0	0	1
Cluster of Glycerophosphodiester phosphodiesterase OS=Triticum aestivum OX=4565 PE=4 SV=1 (A0A3B6PJW8)	A0A3B6PJW8 [2]	81 kDa	0	0	0	0	0	1
Uncharacterized protein OS=Triticum aestivum OX=4565 PE=4 SV=1	A0A3B6QDW0 (+1)	136 kDa	0	0	0	2	0	0
Uncharacterized protein OS=Triticum aestivum OX=4565 PE=3 SV=1	A0A3B6EHB2 (+2)	35 kDa	0	0	0	2	0	0
Uncharacterized protein OS=Triticum aestivum OX=4565 GN=TRAES_3BF077600090CFD_c1 PE=3 SV=1	A0A077RZ13 (+1)	22 kDa	0	2	0	0	0	0
Uncharacterized protein OS=Triticum aestivum OX=4565 PE=3 SV=1	A0A1D5RU47 (+2)	42 kDa	0	0	2	0	0	0
Importin subunit alpha OS=Triticum aestivum OX=4565 PE=3 SV=1	A0A3B5ZQY1 (+1)	59 kDa	0	0	0	2	0	0
A0A3B6CHI9-DECOY	A0A3B6CHI9-DECOY	?	0	2	0	0	0	0
sp|CAS2_BOVIN|	sp|CAS2_BOVIN|	26 kDa	0	2	0	0	0	0
3-isopropylmalate dehydrogenase OS=Triticum aestivum OX=4565 PE=3 SV=1	A0A3B6B8V8 (+3)	42 kDa	0	2	0	0	0	0
